# Divergent Fine-Scale Recombination Landscapes between a Freshwater and Marine Population of Threespine Stickleback Fish

**DOI:** 10.1093/gbe/evz090

**Published:** 2019-04-27

**Authors:** Alice F Shanfelter, Sophie L Archambeault, Michael A White

**Affiliations:** 1 Department of Genetics, University of Georgia; 2 Institute of Ecology and Evolution, University of Bern, Switzerland; 3 Graduate Program in Molecular and Cellular Biology, University of Washington

**Keywords:** recombination, threespine stickleback, linkage disequilibrium, recombination hotspots

## Abstract

Meiotic recombination is a highly conserved process that has profound effects on genome evolution. At a fine-scale, recombination rates can vary drastically across genomes, often localized into small recombination “hotspots” with highly elevated rates, surrounded by regions with little recombination. In most species studied, the location of hotspots within genomes is highly conserved across broad evolutionary timescales. The main exception to this pattern is in mammals, where hotspot location can evolve rapidly among closely related species and even among populations within a species. Hotspot position in mammals is controlled by the gene, *Prdm9*, whereas in species with conserved hotspots, a functional *Prdm9* is typically absent. Due to a limited number of species where recombination rates have been estimated at a fine-scale, it remains unclear whether hotspot conservation is always associated with the absence of a functional *Prdm9.* Threespine stickleback fish (*Gasterosteus aculeatus*) are an excellent model to examine the evolution of recombination over short evolutionary timescales. Using a linkage disequilibrium-based approach, we found recombination rates indeed varied at a fine-scale across the genome, with many regions organized into narrow hotspots. Hotspots had highly divergent landscapes between stickleback populations, where only ∼15% of these hotspots were shared. Our results indicate that fine-scale recombination rates may be diverging between closely related populations of threespine stickleback fish. Interestingly, we found only a weak association of a PRDM9 binding motif within hotspots, which suggests that threespine stickleback fish may possess a novel mechanism for targeting recombination hotspots at a fine-scale.

## Introduction

Meiotic recombination is a highly conserved process across a broad range of taxa (Petes 2001; de Massy 2013). Recombination creates new allelic combinations by breaking apart haplotypes (Otto and Lenormand 2002; Coop and Przeworski 2007), promotes the proper segregation of chromosomes during meiosis in many species (Mather 1936; Kaback et al. 1992; Fledel-Alon et al. 2009), and has a pronounced impact on the evolution of genomes (Maynard-Smith and Haigh 1974; Begun and Aquadro 1992; Charlesworth et al. 1993; Comeron et al. 1999; Duret and Arndt 2008; Webster and Hurst 2012; Mugal et al. 2015; Dapper and Payseur 2017; Kent et al. 2017). In many species, recombination rates can vary dramatically at a fine-scale across a single genome, organized into narrow 1–2-kb “hotspots,” surrounded by large genomic regions with little to no recombination (Jeffreys et al. 1998; Steiner et al. 2002; McVean et al. 2004; Myers et al. 2005; Barton et al. 2008; Baudat et al. 2010; Hellsten et al. 2013). Understanding how recombination rates vary at a fine-scale across species, populations, and even individuals is essential for understanding how this process shapes the molecular evolution of genomes.

In many species, the localization of hotspots is highly conserved across long evolutionary timescales (Tsai et al. 2010; Lam and Keeney 2015; Singhal et al. 2015; Kawakami et al. 2017). For example, finches share upward of 73% of hotspots across 3 Myr of evolution (Singhal et al. 2015), whereas species of *Saccharomyces* share 80% of hotspots over 15 Myr of evolution (Lam and Keeney 2015). Evolutionarily conserved hotspots are generally located around regions of open chromatin such as transcription start sites (TSSs) (Pan et al. 2011; Tischfield and Keeney 2012; Auton et al. 2013; Lam and Keeney 2015; Singhal et al. 2015) and CG-rich regions (i.e., CpG islands) in vertebrates (Campbell et al. 2016; Kawakami et al. 2017). Hotspot localization is thought to be due to the opportunistic nature of SPO11, a meiosis-specific protein which initiates recombination by creating double strand breaks (DSBs) (Romanienko and Camerini-Otero 1999; Celerin et al. 2000) in any region of open chromatin (Pan et al. 2011).

The only documented exception to the strong conservation of recombination hotspots is in mammals, where hotspot location evolves rapidly between closely related species or even between populations (Brick et al. 2012; Pratto et al. 2014; Baker et al. 2015; Smagulova et al. 2016; Stevison et al. 2016). Few hotspots are shared between chimpanzees, bonobos, gorillas, and humans or even subspecies of mice, despite minimum divergence times of hundreds of thousands of years (Brick et al. 2012; Pratto et al. 2014; Stevison et al. 2016). Contrary to the pattern observed in conserved systems, rapidly evolving hotspots typically form away from functional genomic elements and are localized by the zinc finger histone methyltransferase protein, PRDM9 (Baudat et al. 2010; Myers et al. 2010; Parvanov et al. 2010; Brick et al. 2012; Billings et al. 2013; Pratto et al. 2014; Baker et al. 2015; Powers et al. 2016). PRDM9 contains multiple DNA-binding zinc fingers that are under strong positive selection, leading to divergent hotspot localization between closely related species (Oliver et al. 2009; Myers et al. 2010; Billings et al. 2013; Baker et al. 2015). In many species outside of mammals, PRMD9 is missing the complete set of functional domains that are required for localizing recombination hotspots (Baker et al. 2017). Although tremendous progress has been made in characterizing fine-scale recombination rates across the genomes of many species, there is still a relatively limited number of taxa surveyed outside of mammals, making it unclear whether all nonmammalian species will exhibit hotspot conservation at a fine-scale and whether this is always associated with the lack of a functional PRDM9 (Baker et al. 2017). More taxa are needed to elucidate how fine-scale recombination landscapes change over time.

Threespine stickleback fish (*Gasterosteus aculeatus*) are an excellent system to study the evolution of fine-scale recombination rates. Multiple populations of threespine stickleback fish have independently adapted to freshwater environments from marine ancestors in the last 10–15 thousand years, providing the opportunity to study the parallel evolution of hotspots in well-characterized populations across the Northern Hemisphere (Bell and Foster 1994). Broad-scale recombination rates have been examined in threespine stickleback using genetic crosses (Peichel et al. 2001; Roesti et al. 2013; Glazer et al. 2015; Sardell et al. 2018), but fine-scale recombination rates could not be thoroughly estimated due to low marker density or because of small genetic crosses. Threespine stickleback fish are also a useful species to explore whether a nonfunctional PRDM9 is associated with conserved hotspot evolution. Similar to many species outside of mammals (Baker et al. 2017), the threespine stickleback *Prdm9* does not contain a full set of protein domains shown to be important for localizing recombination hotspots (Grey et al. 2017; Imai et al. 2017).

Fine-scale recombination rates can be estimated through a variety of approaches. Recombination rates can be directly measured through genetic linkage maps (Broman et al. 1998; Drouaud et al. 2006; Campbell et al. 2016; Marand et al. 2017) or though sperm genotyping (Jeffreys et al. 2001; Cullen et al. 2002). Both methods require a large number of progeny or sperm and a high density of genetic markers to capture a sufficient number of crossovers. Recombination rates can also be indirectly measured by identifying the binding sites of proteins that initiate DSBs (Smagulova et al. 2011; Pratto et al. 2014) as well as those that repair DSBs through homologous recombination (Froenicke et al. 2002; Dumont and Payseur 2011). Another broadly used approach estimates recombination rates from patterns of linkage disequilibrium (LD) in populations, providing a historical measure of meiotic crossovers over multiple generations (McVean et al. 2004; Myers et al. 2005; Chan et al. 2012; Singhal et al. 2015; Stevison et al. 2016). Although LD-based methods can potentially be biased by demographic history (e.g., bottlenecks, population expansions, and population substructure) (Johnston and Cutler 2012; Dapper and Payseur 2018), these methods offer a powerful approach to estimate recombination rates from multiple populations without the need to construct crosses. In many species, hotspots identified through LD-based methods have been validated using other approaches (Jeffreys et al. 2005; Myers et al. 2006; Morgan et al. 2017).

Here, we used an LD-based approach to estimate genome-wide recombination rates in a marine (Puget Sound) and freshwater (Lake Washington) population of threespine stickleback fish. We found recombination landscapes varied at a fine-scale between the two populations with many regions of elevated rates organized into local recombination hotspots. We found most recombination hotspots were not shared between populations. Although threespine stickleback fish populations have complex demographic histories due to the recent colonization of freshwater environments (Bell and Foster 1994; Hohenlohe et al. 2010; Ferchaud and Hansen 2016; Liu et al. 2016), we found the divergence of recombination hotspots is not completely driven by demography. Among hotspots, there was little evidence of an association with the threespine stickleback annotated PRDM9, indicating hotspots are likely localized by a different mechanism. Our results suggest fine-scale recombination landscapes can diverge over short evolutionary timescales and argue for additional work to understand the diversity in mechanisms that regulate hotspots.

## Materials and Methods

### Population Sampling

Lake Washington threespine stickleback fish were collected near the shore with minnow traps from the north end of the lake (near Kenmore, WA) and the southeast end of the lake (near Mercer Slough, WA). Fish were also collected from open waters at various depths in the middle and northern half of the lake using seine fishing. Puget Sound fish were collected via trawling from open water during a multiday sampling trip in the Whidbey Basin and Bellingham Bay areas of Puget Sound, WA. Fish were sampled from the top 22 m of water.

### Whole-Genome Sequencing and Assembly

Genomic DNA was extracted from caudal fin clips of 13 female and 12 male fish collected from Lake Washington and 18 female and 6 male fish collected from Northern Puget Sound using a standard phenol–chloroform extraction. Libraries were prepared using the Illumina TruSeq kit and were size-selected to target 400-bp fragments. Libraries were multiplexed and paired-end sequencing was performed on an Illumina NextSeq for 300 cycles (Georgia Genomics and Bioinformatics Core, University of Georgia). Residual adapter sequences and low-quality regions were trimmed from the sequencing reads using Trimmomatic (v0.33) with the following parameters: PE –phred 33 slidingwindow:4:20. Trimmed reads were aligned to the revised threespine stickleback reference genome assembly from Bear Paw Lake, AK (supplementary file S5, Supplementary Material online, https://datadryad.org/resource/doi:10.5061/dryad.q018v/1; last accessed March 2017. Glazer et al. 2015) using Bowtie2 (v2.2.4, default parameters) (Langmead and Salzberg 2012). With these parameters, the average alignment rate for Lake Washington was 94.2% and 87.3% for Puget Sound. Reads with a mapping PHRED quality score of 20 or less were removed from the analysis (Samtools, v1.2.0, default parameters; Li et al 2009). For Puget Sound, four female individuals had 5× or lower sequencing coverage and were removed from the analysis, resulting in a final sample size of 20 fish. After removing poorly aligned reads and low-coverage individuals, the average read coverage across all individuals in each population was 17× and 22× for Lake Washington and Puget Sound, respectively.

Two outgroup species were used to infer ancestral allele states and to estimate mutation matrices for each population (see Estimation of Recombination Rates). Whole-genome Illumina sequences for one female ninespine stickleback fish (*Pungitius pungitius*, DRX012173) (White et al. 2015) and one female blackspotted stickleback fish (*Gasterosteus wheatlandi*, DRX012174) (Yoshida et al. 2014) were aligned to the revised threespine stickleback genome assembly (Glazer et al. 2015) using Bowtie2 (v2.2.4). Less stringent alignment parameters were used to allow for greater sequence divergence between threespine stickleback and each outgroup (*-*D 20 –R 3 –N 1 –L 20 –I S, 1, 0.50 -rdg 3, 2 -rfg 3, 2 -mp 3). The overall alignment rate of *P. pungitius* was 46.0%, whereas the overall alignment rate of *G. wheatlandi* was 74.2%. The higher alignment rate of *G. wheatlandi* is consistent with *G. wheatlandi* sharing a more recent common ancestor with *G. aculeatus* (Kawahara et al. 2009).

### Single Nucleotide Polymorphism Genotyping

Single nucleotide polymorphisms (SNPs) were genotyped in each threespine stickleback population and outgroup species independently following the GATK best practices for SNP discovery for whole-genome sequences (v3.6) (Van der Auwera et al. 2013). Polymerase chain reaction duplicates were removed using MarkDuplicates (REMOVE_DUPLICATES=true). Regions around insertions or deletions (indels) were realigned with RealignerTargetCreator (default parameters) and IndelRealigner (default parameters). Variants were called for each individual using HaplotypeCaller (genotyping mode DISCOVERY). Joint genotyping (GenotypeGVCFs, default parameters) was completed by pooling all individuals for each population. Low-quality SNPs were filtered from the data set using vcftools (v0.1.12b) (Danecek et al. 2011). Sites were removed if they had more than two alleles, if genotypes were missing in any of the individuals, or if genotype quality scores were <30. To prevent bias from high copy number variants or poorly sequenced regions, sites were also removed if the population mean depth coverage was less than half or greater than twice the average coverage for each population (Lake Washington: 8×–34× read-depth coverage; Puget Sound: 11×–44× read-depth coverage). Singletons and sites fixed for the alternate allele across all individuals in a population were also removed. After filtering, the Lake Washington population had 5,054,729 SNPs genome-wide (11 SNPs/kb) and the Puget Sound population had 4,142,876 SNPs (9 SNPs/kb) genome-wide (prior to filtering Lake Washington had 11,937,220 SNPs and Puget Sound had 11,070,421 SNPs). For the outgroup species, *P. pungitius* and *G. wheatlandi*, low-quality SNPs were excluded by removing variants with a genotyping quality score <30 or a read depth ≤2, resulting in 13,691,521 SNPs genome-wide in *G. wheatlandi* (16,783,618 SNPs prior to filtering) and 7,791,420 in *P. pungitius* (26,173,287 SNPs prior to filtering). To test whether analyses were robust to the SNP read-depth filters, over- and under-filtered SNP sets were also tested across a single representative autosome, chromosome one. The overfiltered SNP set used a read-depth range of 13×–23× in Lake Washington and 17×–29× in Puget Sound. The underfiltered SNP set used a read-depth range of 6×–51× in Lake Washington and 7×–66× in Puget Sound.

### Population Structure

Population substructure within Puget Sound and Lake Washington was investigated using FastStructure (v1.0) (Raj et al. 2014). Only biallelic sites with no missing data were retained for each population and the sex chromosomes were excluded. The final data sets consisted of 4,824,791 SNPs for Lake Washington and 3,876,608 SNPs for Puget Sound. For both populations, five trials of *K* were completed at *K* values of 1–5 where a *K* of 1 would indicate no population substructure and a *K* of 5 would indicate that individuals were likely from several different populations. The model that best explained the population structure for Lake Washington and Puget Sound was determined using chooseK.py (Raj et al. 2014) and the structure plot was visualized using distructK.py (Raj et al. 2014).

Genetic admixture between the two populations was investigated using a similar approach with FastStructure. Biallelic SNPs without missing data from both populations were merged using vcftools (Danecek et al. 2011). The final SNP data set was composed of 4,113,937 SNPs. Three trials were completed at *K* values of 1–3. These *K* values were chosen to differentiate scenarios where Lake Washington and Puget Sound were one panmictic population (*K* = 1) or Lake Washington and Puget Sound were two distinct populations (*K* = 2). A *K* of 3 was chosen to identify any hidden population structure within either population. The model that best explained the population structure was determined using chooseK.py (Raj et al. 2014).

Population structure between and within Lake Washington and Puget Sound was also explored using a principal component analysis on male and female fish separately. SNPs from the male and female data sets were pruned using Plink (–maf 0.01 –indep-pairwise 50 5 0.2) (Purcell et al. 2007). The final SNP data set included 3,983,008 SNPs for males and 4,039,016 SNPs for females. Principal components were calculated using Plink.

### Haplotype Phasing

Each chromosome was phased independently with SHAPEIT (v2.r837), a read-aware phasing tool (Delaneau et al. 2013). Illumina reads that contained at least two heterozygous SNPs were identified as phase-informative reads and used to more accurately phase haplotypes. Only reads with a mapping quality score >20 were used as phase-informative reads. Convergence of the Markov chain Monte Carlo (MCMC) algorithm was estimated by examining switch error rates between individual runs. A low switch error rate would indicate that the MCMC phasing runs have converged on a similar haplotype configuration. Switch error was measured using vcftools (v0.1.12b) using –diff-switch-error (Danecek et al. 2011). A low switch error was achieved within a reasonable run time with the following SHAPEIT parameters: –main 2000 –burn 200 –prune 210 –states 1000 (average switch error between phasing runs: 0.824% for Lake Washington and 1.26% for Puget Sound). All other parameters were left at the default values.

### Estimation of Recombination Rates

Recombination rates were estimated with LDHelmet (v1.7; Chan et al. 2012). LDHelmet estimates historical recombination rates from population data by analyzing patterns of LD across phased haplotypes. LDHelmet was chosen for rate estimation because LDHelmet can handle higher SNP densities, does not assume neutral evolution across the genome, and allows the incorporation of quadra-allelic mutation models and ancestral allele state priors to improve rate estimation (Chan et al. 2012). Threespine stickleback fish have a high SNP density (∼1 SNP per 100 bp), have ancestral allele information from outgroup species, and the genome has potentially been impacted by selection as marine and freshwater populations have adapted to new environments. Although LDHelmet was originally designed for *Drosophila*, it has recently been applied to a diverse group of species, including other populations of threespine stickleback fish (Singhal et al. 2015; Booker et al. 2017; Kawakami et al. 2017; Sardell et al. 2018).

The ancestral allele state was defined for every SNP in each threespine stickleback population by comparing to the allele present in the two outgroup species. An ancestral allele state could not be assigned if a polymorphism was segregating among the outgroup species. Therefore, SNPs were only assigned an ancestral state if *P. pungitius* and *G. wheatlandi* were homozygous for the same allele. The ancestral allele was assumed to be the nucleotide carried by *P. pungitius* and *G. wheatlandi* and was assigned a prior probability of 0.91. To allow for uncertainty in the ancestral allele state, the other three possible nucleotides were assigned prior probabilities of 0.03. If the ancestral allele state could not be inferred, the prior probability of each nucleotide being the ancestral allele was set as the overall frequency of that particular nucleotide on the chromosome. Nucleotide frequencies were empirically determined from all sites on a threespine stickleback chromosome where *P. pungitius* and *G. wheatlandi* had read coverage that passed the filtering scheme. Mutation matrices were estimated for each population separately. For every position where an ancestral allele state could be inferred, the total number of each type of mutation away from the ancestral allele was quantified. A normalized 4 × 4 mutation matrix was generated for each chromosome as previously described (Chan et al. 2012). The ancestral allele state and mutation matrices were generated using a custom Perl script.

Custom Python scripts were used to create the SNP sequence and SNP position input files for LDHelmet. Full FASTA sequences were created using vcf2fasta from vcflib (available at https://github.com/vcflib/vcflib/; last accessed August 2016). Each LDHelmet module was run using the following parameters. Haplotype configuration files were created for each chromosome with the find_confs module using a window size of 50 SNPs (-w 50). Likelihood lookup tables were created using table_gen with the recommended grid of population-scaled recombination rates per base pair (*ρ*/bp) (-r 0.0 0.1 10.0 1.0 100.0). Watterson’s *θ* was estimated using a custom Python script with the R package, PopGenome (Pfeifer et al. 2014), where Watterson’s *θ* was calculated in 2-kb regions with a sliding window of 1 kb and all windows were averaged together. To maintain a reasonable computational time, a single representative likelihood lookup table was generated for the autosomes of each population from chromosome one, using the average Watterson’s *θ* between Lake Washington and Puget Sound (-t 0.002). Although Watterson’s *θ* was different between the Lake Washington and Puget Sound populations, previous studies have determined that small changes to parameters such as Watterson’s *θ* do not affect the final likelihoods (McVean et al. 2004; Auton and McVean 2007; Stukenbrock and Dutheil 2018). Separate likelihood lookup tables were created for the pseudoautosomal region (PAR) of the sex chromosomes (chromosome 19). Padé coefficient files were created using the module pade with a Watterson’s *θ* of 0.002 and the recommended 11 padé coefficients (-t 0.002 –x 11). The module rjmcmc was run for 1 million iterations with 100,000 burn in iterations, a block penalty of 10, and a window size of 50 SNPs (-w 50 –b 10 –burn_in 100000 –n 1000000). Population-scaled recombination rates were extracted from the rjMCMC run with the post_to_text module. Recombination rates were reported in *ρ*/bp where *ρ* is a population-scaled recombination rate (4*N*_e_*r*).

### Correlation with Genetic Maps

Population-scaled recombination rates were compared with recombination rates estimated from a high-density genetic linkage map (wild caught marine male from Little Campbell River (British Columbia, Canada) × wild caught freshwater female from Fishtrap Creek (Washington, USA) (Glazer et al. 2015). Recombination rates from LDHelmet were converted from *ρ*/bp to cM/Mb as previously described (Smukowski Heil et al. 2015). Briefly, the recombination rate (cM/Mb) was calculated between every pair of adjacent markers in the genetic map and a chromosome-wide recombination rate was calculated as the average among the regions. The average LD-based recombination rate (*ρ*/Mb) was computed in the same individual regions of a chromosome in Lake Washington and Puget Sound by averaging the per bp rho estimate across the total length of the region (*ρ*/Mb). A single conversion factor was calculated for each chromosome. Each conversion factor was calculated by dividing the average linkage map recombination rate for a chromosome (in cM/Mb) by the average LD-based recombination rate (*ρ*/Mb) for that chromosome.

### Identification of Recombination Hotspots

Recombination hotspots were defined using a sliding-window approach. In each window, the average recombination rate within a 2-kb window was compared with the average recombination rate from the 40-kb regions flanking either side of the 2-kb window. Hotspots were defined as the 2-kb regions that had a 5-fold or higher recombination rate relative to the mean recombination rate in the flanking background regions. The 2-kb windows iterated forward in 1-kb increments. If multiple hotspots were found within a 5-kb region, only the hotspot with the highest rate was retained. Errors in the reference genome assembly could generate false hotspots. To limit this, all hotspots that spanned a contig boundary in the reference genome were removed (394 hotspots out of 4,659 total hotspots). Hotspots were considered shared between populations if the midpoints of the two hotspots were within 3 kb of each other. Random permutations were used to calculate the expected amount of hotspot overlap between Lake Washington and Puget Sound. Ten thousands random permutations were drawn from the genome totaling the number of 2-kb hotspots for each population. Recombination hotspots were identified and filtered using custom Perl and Python scripts. Each hotspot was matched to a randomly selected 2-kb coldspot, which was located at least 25 kb from any identified hotspot, contained a GC nucleotide content that was within 2% of the hotspot after removing ancestral CpG sites (GC-matched), and had a mean recombination rate that was less than half the background recombination rate of the population (Lake Washington: <0.017 *ρ*/bp; Puget Sound: <0.035 *ρ*/bp). The total number of phase-informative reads from SHAPEIT was counted in hotspots and coldspots. The proportion of phase-informative reads compared with the total number of reads at each hotspot or coldspot was calculated using custom Python scripts.

### Estimating Recombination Rates across the X Chromosome

Population-scaled recombination rate was estimated across the X chromosome using only females (Lake Washington: 13 individuals; Puget Sound: 14 individuals). Females were only used for this analysis because of residual sequence homology between the X and Y chromosome in threespine stickleback fish makes differentiating X-linked and Y-linked SNPs difficult in males (White et al. 2015). LDHelmet was run with X chromosome-specific mutation matrices, ancestral allelic states, and likelihood lookup tables derived females only. The average recombination rate across the X chromosome, including and excluding the PAR, was calculated for both populations. Any observed differences in recombination rate between the X chromosome and autosomes could partially be driven by the smaller total sample size. To test whether overall sample size had an effect on recombination rates estimated by LDHelmet, chromosome one was randomly downsampled to equal the total number of females in each population. Recombination rates were estimated with LDHelmet as previously described for five randomly downsampled trials. Average recombination rate was estimated in 500-kb windows across chromosome one. Rates in each window were compared with those estimated from the full sample set. Custom Python scripts and R were used to calculate the average rates.

### Genetic Variation within and between Populations

Within population nucleotide diversity (*π*) and Tajima’s *D* were calculated separately for each chromosome. To capture rare variants, previously excluded singletons were included in the analysis. Nucleotide diversity and Tajima’s *D* were calculated in 2-kb windows across each chromosome using the R package, PopGenome (Pfeifer et al. 2014) and a custom Python script. Total *π* among both populations was calculated in 2-kb windows from the combined set of SNP variants of each population. To estimate genetic differentiation between Lake Washington and Puget Sound, *F*_ST_ was calculated using vcftools (–weir-fst-size 1000; Danecek et al 2011). Hotspots and coldspots (each 2 kb in length) were compared against the rest of the genome (split into 2-kb regions, excluding hotspots and coldspots). Statistical significance was assessed by randomly drawing a subset of 2-kb regions from the genome-wide sample, equal in size to the number of coldspots or hotspots. For each random sample, the median *F*_ST_ or *π* was calculated. Ten thousand samples were drawn and statistical significance was estimated under the null hypothesis that the observed *F*_ST_ or *π* of coldspots or hotspots was from the same distribution as the genome-wide random samples.

### Estimation of Demographic History

Demographic history can affect LD-based estimates of recombination rates (Johnston and Cutler 2012; Dapper and Payseur 2018). To determine whether the demographic history of threespine stickleback fish could influence the ability to detect recombination hotspots, hotspots were assayed in simulated haplotypes with known recombination profiles and demographic histories. Demographic histories used in the simulations were based on the estimated histories of Lake Washington and Puget Sound, modeled using a Pairwise Sequentially Markovian Coalescent (PSMC) process with default parameters (Li and Durbin 2011; Liu and Hansen 2017). PSMC was run on all individuals from both populations and confidence intervals were estimated on 100 bootstrap replicates. One female from each population was used for predicting demographic histories for simulations. Demographic histories were visualized using psmc_plot.pl (Li and Durbin 2011).

### Simulations Using Estimated Demographic Histories

Two hundred fifty kilobase haplotypes with four 2-kb recombination hotspots were simulated using the program fin, part of the LDHat software package (McVean et al. 2004; Auton and McVean 2007). The hotspots were placed 50 kb apart at 75, 125, 175, and 225 kb. The background recombination rate was set at 0.03 *ρ*/kb. Hotspots had varied intensities from 2 to 20 times the background rate, set at 0.06, 0.15, 0.3, and 0.6 *ρ*/kb. One scenario simulated a constant effective population size, with 500 sequences, 40 haplotypes each, with an average Watterson’s *θ* of 0.00355, the average between Lake Washington and Puget Sound (–nsamp 40 –len 250000 –theta 0.00355). For both populations, a bottleneck was simulated 8,000 generations ago (Puget Sound: *t* = 0.029, theta = 0.0036; Lake Washington: *t* = 0.022, theta = 0.0035). Two bottleneck strengths were simulated by setting the probability that a lineage coalesces to 10% or 90% (*s* = 0.1, 0.9). These strengths represent two extreme bottleneck scenarios that fell outside of the observed bottlenecks estimated by PSMC (Lake Washington had a 28.94% reduction in population size; Puget Sound had a 44.05% reduction in population size). Overall, hotspot sharing between simulated Lake Washington and simulated Puget Sound populations was quantified by examining all pairwise comparisons between populations and bottleneck strengths. The number of false positive and false negative hotspots was calculated using custom Python scripts.

### Location of Hotspots around TSSs

Transcript annotations from Ensembl (build 90) were lifted to the revised threespine stickleback genome assembly (Glazer et al. 2015) by aligning each transcript using BLAT (v36, default parameters; Kent 2002). Aligned transcripts were only retained if the entire transcript aligned to the revised genome assembly. Transcript start sites (TSSs) consisted of a 6-kb region, centered at the start position of the transcript. A hotspot was considered overlapping with a TSS if the midpoint of the hotspot overlapped with any part of a 6-kb TSS region. Enrichment of hotspots in TSSs were compared against 10,000 random permutations. Six-kilobase regions were randomly drawn across the genome, totaling the number of hotspots identified in each population. TSS annotation filtering, overlap of hotspots with TSSs, and random permutations were completed using custom Python scripts.

### Gene Ontology Analysis

Gene ontology (GO) terms associated with genes within 3 kb of hotspots were analyzed using custom python scripts. Gene IDs and GO terms were downloaded from Ensembl using Biomart (Smedley et al. 2015). The total number of occurrences for each GO term was calculated within shared hotspots, Lake Washington population-specific hotspots, and Puget Sound population-specific hotspots. GO term enrichment in each hotspot set was compared against 10,000 random permutations of the same sized set drawn randomly from across the genome (excluding original hotspots). *P* values were adjusted for multiple testing using a Bonferroni correction based on the total number of GO terms in each hotspot set (shared hotspots: 118 GO terms; Lake Washington population-specific hotspots: 605 GO terms; Puget Sound population-specific hotspots: 869 GO terms).

### GC-Biased Substitutions

GC to AT and AT to GC substitutions were quantified within all 2-kb recombination hotspots combined and coldspots combined. The equilibrium GC content was calculated as the proportion of AT to GC substitutions out of the total pool of substitutions (AT to GC and GC to AT) (Sueoka 1962; Meunier and Duret 2004; Singhal et al. 2015). To increase the total number of sites available for the analysis, the ancestral allele state was inferred using only *G. wheatlandi*, rather than requiring a matching ancestral allele in both *G. wheatlandi* and *P. pungitius.* Because CpG sites can have higher mutation rates (Fryxell and Moon 2005; Weber et al. 2014), all consecutive CG sites in the ancestral sequence were removed from the analysis. Equilibrium GC content of hotspots and coldspots was compared with the remaining 2-kb regions across the genome after hotspots and coldspots were removed.

### DNA Motif Identification

MEME (v4.11.0) was used to identify novel DNA motifs enriched in hotspots and matched coldspots (Bailey and Eklan 1994). MEME ignored motif occurrences if they were present in a hotspot multiple times (-mod zoops). This was to prevent the reporting of repetitive motifs. MEME was run separately for each chromosome and population and terminated when 50 motifs were identified (-nmotifs 50). Motif identification was conducted separately for shared hotspots and population-specific hotspots.

The DNA-binding protein, PRDM9, is important for localizing recombination hotspots in mammals (Baudat et al. 2010; Myers et al. 2010; Parvanov et al. 2010; Brick et al. 2012; Billings et al. 2013; Pratto et al. 2014; Baker et al. 2015; Powers et al. 2016). To determine if any PRDM genes had a role in localizing hotspots in threespine stickleback fish, FIMO (v4.11.0, default parameters; Grant et al. 2011) was used to scan hotspot sequences for the predicted DNA-binding motifs for each of the 11 annotated *Prdm* genes in the threespine stickleback genome (Ensembl, build 90). DNA-binding motifs for each PRDM protein were predicted using the Cys_2_His_2_ zinc finger prediction tool, Predicting DNA-binding specificities for the Cys_2_His_2_ zinc finger proteins (Persikov et al. 2009; Persikov and Singh 2014). Predicted zinc finger domains were included if the HMMER bit score for the zinc fingers was 17.7 or higher (Persikov et al. 2009; Persikov and Singh 2014). To determine the expected number of occurrences of a motif of the same length and GC composition in hotspots, the PRDM motifs were shuffled 100 separate times. FIMO was run on the shuffled motifs to create a null distribution. Motifs were shuffled using a custom python script.

## Results

### Lake Washington and Puget Sound Are Genetically Distinct Populations

Freshwater populations of threespine stickleback fish frequently exhibit signs of past bottlenecks, consistent with their colonization from marine ancestors ∼10–15 thousand years ago (Bell and Foster 1994). Given the recent divergence and the close geographic proximity between Lake Washington (freshwater) and Puget Sound (marine), we first examined whether these two populations were genetically distinct. Using FastStructure, a two population model was the most highly supported (marginal likelihood: −0.834, supplementary fig. 1, Supplementary Material online). Because samples were collected from multiple locations within Puget Sound and Lake Washington, we also investigated whether either population had evidence of substructure. Previous sampling from Puget Sound did not reveal population substructure, but some localities within Lake Washington have shown some structure (Kitano et al. 2008). From our sampling, FastStructure only supported a single population for Lake Washington (*K* = 1; marginal likelihood: −0.910) and Puget Sound (*K* = 1; marginal likelihood: −0.937). Principal component analysis of both populations confirmed the results that Puget Sound and Lake Washington are genetically distinct clusters (supplementary fig. 2, Supplementary Material online) with no observable population substructure in either population.

Within each population, we explored whether there were signatures of past bottleneck events. The average nucleotide diversity within both populations was similar (Lake Washington: 0.0032; Puget Sound: 0.0028), whereas the genome-wide average nucleotide diversity between populations was 0.0037. The nucleotide diversity values we calculated are similar to previously reported values for other marine and freshwater stickleback populations (Kitano et al. 2007; Hohenlohe et al. 2010; Guo et al. 2015). Both populations had negative Tajima’s *D* values (Tajima 1989), consistent with an excess of rare variants from a recent population expansion (Lake Washington: −0.422; Puget Sound: −0.723).

**Fig. 1.**
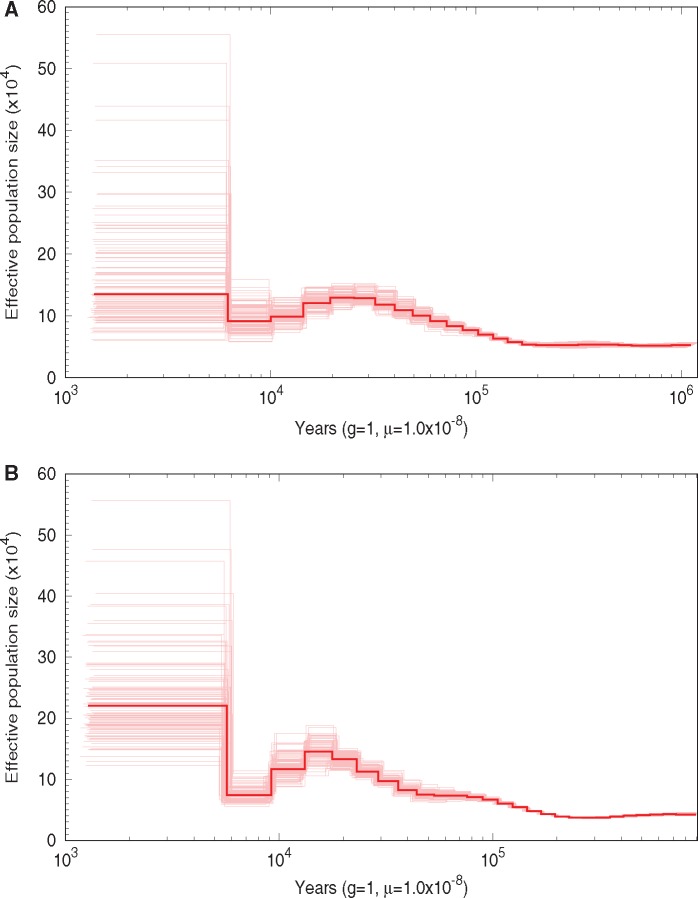
—Lake Washington and Puget Sound have experienced past population bottlenecks. Demographic history for Lake Washington (*A*) and Puget Sound (*B*) was estimated using PSMC from a single female fish from each population. One hundred bootstrap replicates around the estimated history are shown.

The demographic histories of Lake Washington and Puget Sound were estimated using PSMC models (fig. 1 and supplementary fig. 3, Supplementary Material online). Puget Sound experienced a bottleneck from around 18,000 years ago until about 8,000 years ago where the effective population size decreased to 74,250 ± 1,259 individuals (starting *N*_e_: 132,700 ± 796), whereas Lake Washington experienced a small bottleneck around the same time where the effective population size decreased to 91,760 ± 1,960 individuals (starting *N*_e_: 129,138 ± 897) (fig. 1). Both populations have had a constant effective population size for the last ∼5,000 years. Puget Sound has a larger effective population size than Lake Washington, matching the expected pattern of marine populations having larger effective population sizes than freshwater populations (Gow et al. 2006; Makinen et al. 2006; DeFaveri and Merila 2015).

### Broad-Scale Recombination Rates Are Highly Correlated between Populations

Using a dense set of SNP markers from whole-genome sequencing, we estimated recombination rates across the genomes of Lake Washington and Puget Sound threespine stickleback fish. The average genome-wide population recombination rate in Lake Washington was half of the rate observed in Puget Sound (Lake Washington: 0.035 *ρ*/bp; Puget Sound: 0.072 *ρ*/bp; Wilcoxon rank test; *P* < 0.001, supplementary table 1, Supplementary Material online). Overall read alignment of Puget Sound was lower than the Lake Washington population (see Materials and Methods), which may have affected our overall ability to detect SNPs in Puget Sound. To test whether SNP density may have caused the observed difference in genome-wide recombination rate between the two populations, we altered the read-depth filters to over- or under-filter the SNP set in each population. Puget Sound retained a roughly 2-fold higher recombination rate compared with Lake Washington with the over- or under-filtered SNP sets (Wilcoxon rank test; *P* < 0.001 for all over- or under-filtered SNP set comparisons; supplementary table 2, Supplementary Material online). Because Lake Washington had a greater number of fish than the Puget Sound population, we also explored whether overall sample size had an effect on our estimates of recombination rate. We randomly down sampled Lake Washington for chromosome one to a sample size equal in number to Puget Sound and found Puget Sound still retained a roughly 2-fold higher recombination rate (Lake Washington complete set of 25 individuals: 0.025 *ρ*/bp; Lake Washington downsampled set of 20 individuals: 0.027 *ρ*/bp; Puget sound complete set of 20 individuals: 0.061 *ρ*/bp; Wilcoxon rank test; *P* < 0.001).

**Fig. 2.**
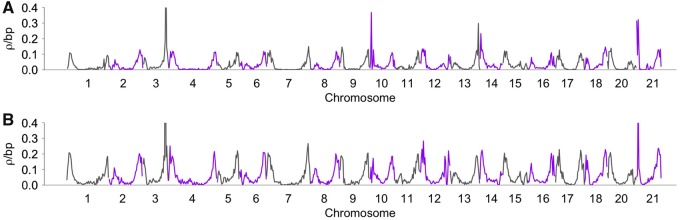
—Recombination rates are similar at a broad scale in each population. Mean recombination rates were estimated using LDHelmet in nonoverlapping 500-kb windows for each autosome in (*A*) Lake Washington and (*B*) Puget Sound. Centromere positions are shown in supplementary figures 6 and 7, Supplementary Material online. Transitions between gray and purple indicate different chromosomes.

Despite having an overall lower genome-wide recombination rate in Lake Washington, recombination rates were largely correlated at broad scales between the two populations. We observed a highly significant positive correlation of recombination rates between the populations at the scale of 500-kb windows (Spearman’s rank correlation; rho = 0.931, *P* < 0.001; fig. 2; supplementary fig. 4, Supplementary Material online). To test whether these correlations were robust to sample size differences between Puget Sound and Lake Washington, SNP filtering, and the use of a prior distribution over ancestral alleles, we estimated recombination rates across chromosome one under multiple filtering scenarios. These correlations held regardless of filtering scheme (Spearman’s rank correlation; *P* < 0.001; supplementary fig. 5, Supplementary Material online) and sample size of Lake Washington (Lake Washington downsampled set of 20 individuals vs. Puget Sound Complete set of 20 individuals; Spearman’s rank correlation; rho = 0.907; *P* < 0.001). Additionally, recombination rates were lower at the center of chromosomes (center 25% of all chromosomes) and significantly higher at the terminal ends of chromosomes (terminal 25% of all chromosomes) for both populations (Wilcoxon rank test; Lake Washington terminal ends: 0.069 *ρ*/bp; Lake Washington center of chromosomes: 0.009 *ρ*/bp; *P* < 0.001; Puget Sound terminal ends: 0.108 *ρ*/bp; and Puget Sound center of chromosomes: 0.016 *ρ*/bp; *P* < 0.001; fig. 2). Rate differences at terminal chromosome ends have been documented in other populations of threespine stickleback (Roesti et al. 2013; Glazer et al. 2015; Sardell et al. 2018) as well as across a wide-range of other animals, plants, and fungi (Broman et al. 1998; See et al. 2006; Barton et al. 2008; Berner and Roesti 2017).

**Fig. 3.**
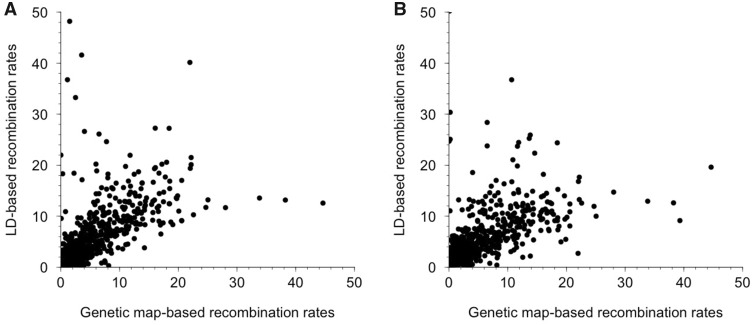
—LD-based estimates of recombination rates are highly correlated with estimates from genetic linkage maps. Population-scaled recombination rates were converted to cM/Mb. There is a significant positive correlation between LD-based recombination rates and genetic map-based recombination rates in (*A*) Lake Washington (Spearman’s rank correlation; rho = 0.830; *P* < 0.001) and (*B*) Puget Sound (Spearman’s rank correlation; rho = 0.810; *P* < 0.001).

To determine whether the broad-scale recombination rates we estimated from LD-based methods are concordant with recombination rates measured from linkage mapping, we compared the rates from Lake Washington and Puget Sound with the rates estimated from a previously constructed genetic linkage map (Glazer et al. 2015). We found a significant positive correlation between recombination rates in both populations and the linkage map (Spearman’s rank correlation; Lake Washington: rho = 0.830, *P* < 0.001; Puget Sound: rho = 0.810, *P* < 0.001; fig. 3). These data indicate that broad-scale changes are conserved across multiple populations of threespine stickleback fish and confirm that the recombination rates estimated from LD-based methods largely parallel the rates observed from genetic linkage maps.

### Hotspot Locations Are Divergent between Threespine Stickleback Populations

**Fig. 4.**
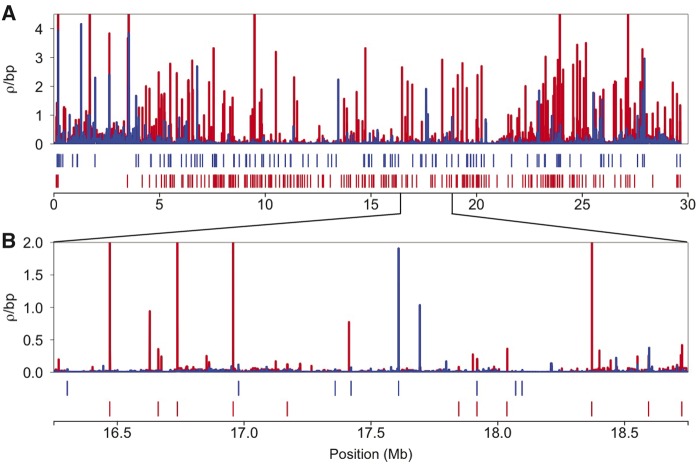
—Recombination rates vary at a fine-scale across chromosome one. (A) Population-scaled recombination rates across chromosome one are shown for Puget Sound (red) and Lake Washington (blue). (B) A subset of chromosome one is shown to highlight population-specific peaks of recombination across a narrow 2.5-Mb region. Only recombination rates below 4.5 *ρ*/bp are shown. Tick marks below each chromosome indicate the location where hotspots were identified. The remaining chromosome plots are in supplementary figures 6 and 7, Supplementary Material online.

**Fig. 5.**
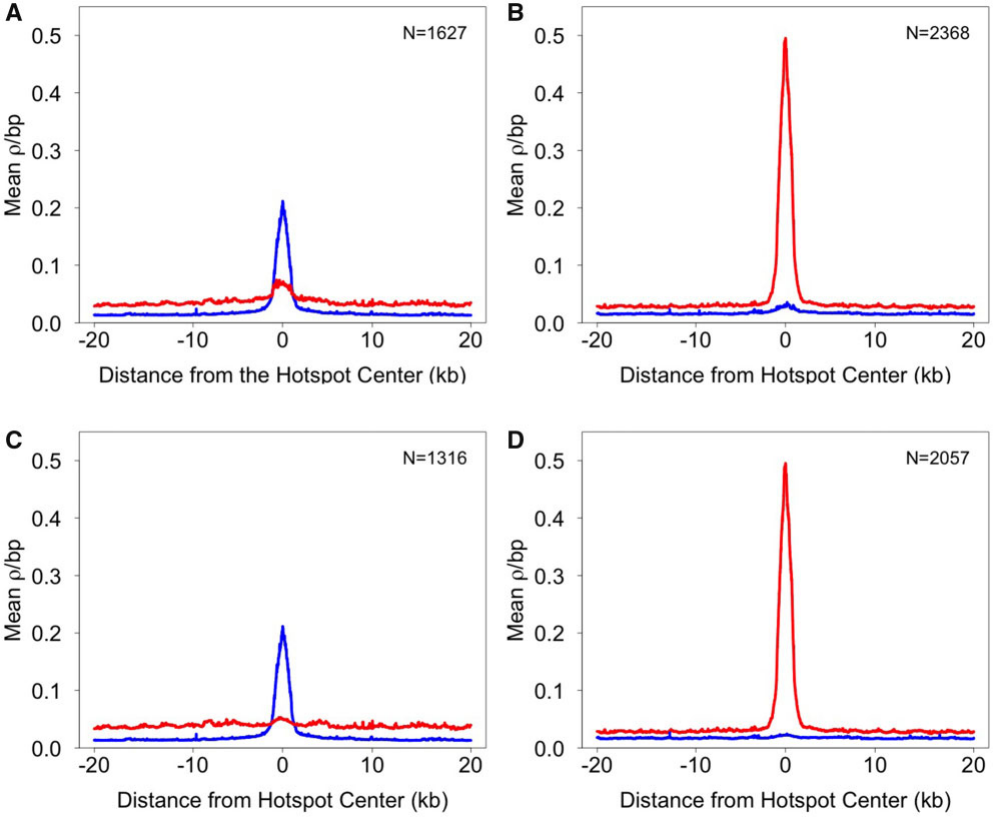
—LD-based recombination rates around hotspots are population-specific. Mean recombination rates are shown across a 40 kb interval, flanking the center of hotspots. (A) The mean recombination rate in shared and population-specific Lake Washington hotspots is higher in the Lake Washington population (blue) compared with the same loci in the Puget Sound population (red). (B) The mean recombination rate in shared and population-specific Puget Sound hotspots is higher in the Puget Sound population compared with the same loci in the Lake Washington population. (C and D) The pattern is more pronounced when shared hotspots are removed from the comparison, leaving only the population-specific hotspots.

Although broad-scale (Mb) recombination rates tend to be conserved over longer evolutionary timescales (Kong et al. 2002; Serre et al. 2005; Fledel-Alon et al. 2009; Stevison et al. 2016), fine-scale (kb) rates within chromosomes can rapidly evolve (McVean et al. 2004; Myers et al. 2005; Barton et al. 2008; Hellsten et al. 2013). Consistent with this, we found highly variable fine-scale recombination rates across individual chromosomes in both Lake Washington and Puget Sound (fig. 4; supplementary figs. 6 and 7, Supplementary Material online). Using a sliding-window approach, we identified 2,368 hotspots in Puget Sound and 1,627 hotspots in Lake Washington. Strikingly, only 311 of these hotspots were shared between populations (13.1% of hotspots in Puget Sound and 19.1% of hotspots in Lake Washington). This lack of hotspot overlap between Lake Washington and Puget Sound may, in part, be due to hotspots falling just below the hotspot threshold. To investigate this, we looked for any increase in recombination rate at loci where hotspots were present in one population, but absent in the other. We found little evidence of a localized increase in recombination rate in these regions. Recombination rates were close to the background rate at the same loci in the population where hotspots were deemed absent (fig. 5*A* and *B*). This pattern was even more apparent when shared hotspots were removed from the analysis (fig. 5*C* and *D*). The small degree of overlap we observed in hotspots between the populations was much greater than what would be expected from chance alone (10,000 random permutations; *P* < 0.001; supplementary fig. 8, Supplementary Material online), indicating much of the hotspot overlap likely represents shared ancestry. We also explored whether shared hotspots exhibited recombination rates different from population-specific hotspots. On average, shared hotspots had lower recombination rates than population-specific hotspots (Lake Washington: shared hotspots: 0.086 *ρ*/bp; population-specific hotspots: 0.134 *ρ*/bp; Puget Sound: shared hotspots: 0.163 *ρ*/bp; population-specific hotspots: 0.283 *ρ*/bp).

Hotspot divergence between populations could partially be driven by errors in haplotype phasing. Incorrectly phased heterozygous sites can artificially increase local rates of recombination, leading to false positives. To increase accuracy, we used a phasing program that incorporates phase-informative reads (SHAPEIT; Delaneau et al. 2013). Therefore, phasing should be most accurate in regions of the genome where pairs of heterozygous SNPs are linked within single paired-end reads. If hotspots are largely caused by errors in phasing, we may expect these regions to have a deficiency of phase-informative reads within each population. Instead, we found over half of SNP pairs among coldspots and hotspots in Lake Washington (hotspots: 66.6%; coldspots: 63.6%) and Puget Sound (hotspots: 61.6%; coldspots: 50.6%) were supported by at least one phase-informative read, suggesting the differences in recombination rates between coldspots and hotspots is not due to a deficiency of known phasing information between SNPs.

We also tested whether fine-scale recombination rates were influenced by population sample size, by our SNP filtering scheme, or were sensitive to the use of a prior distribution over ancestral alleles. In each case, we found the recombination rate among hotspots in the observed set across chromosome one to be highly correlated to the fine-scale recombination rates at the same loci in the over- or under-filtered SNP trials (Spearman’s rank correlation; *P* < 0.001 for the over- and under-filtered SNP sets; supplementary figs. 10 and 11, Supplementary Material online), in the trial where sample size was reduced in Lake Washington (Spearman’s rank correlation; rho = 0.974; *P* < 0.001), and when an ancestral prior distribution was not used (*P* < 0.001; supplementary fig. 12, Supplementary Material online). Furthermore, we also tested whether inclusion of a small number of SNPs that deviated from Hardy–Weinberg equilibrium influenced fine-scale recombination rates (Lake Washington: 3.74% SNPs deviated from equilibrium; Puget Sound: 2.80% SNPs deviated from equilibrium). Nonequilibrium SNPs did not appear to have a major impact on our recombination rate estimates. Recombination rates at hotspots in the observed set of chromosome one were highly correlated with rates at the same loci in the trial where nonequilibrium SNPs were removed (*P* < 0.001; supplementary fig. 13, Supplementary Material online). Combined, these results indicate our detection of hotspots is largely robust to many of the filters we applied in our analysis.

### Demographic History Does Not Completely Account for Hotspot Divergence

To explore how changes in past effective population size (*N*_e_) may have affected our ability to detect hotspots, we simulated haplotypes with known demographic histories that were similar to those estimated from Lake Washington and Puget Sound, along with a known distribution of recombination hotspots. If the minimal hotspot overlap we observed between populations of threespine stickleback fish was because of high false positive and false negative rates induced by demographic history, we would expect hotspots to be incorrectly called to a similar degree in the bottleneck simulations. Both bottleneck strengths exhibited elevated false positive and false negative rates compared with the control simulation, with the highest false positive and false negative rates under the strong bottleneck scenario (supplementary table 3, Supplementary Material online). To determine the overall effect of elevated error rates on determining the number of shared hotspots between populations, we compared the simulated Lake Washington haplotypes to the simulated Puget Sound haplotypes from both bottleneck scenarios. Despite the elevated error rates, hotspot sharing was higher between the simulated populations than the observed number of hotspots shared between actual Lake Washington and Puget Sound populations for the weak bottleneck (weak bottleneck: Lake Washington: 59.7%; Puget Sound: 55.2%; actual Puget Sound shared hotspots: 13.1%; actual Lake Washington shared hotspots: 19.1%). This indicates that a weak bottleneck in both populations is not sufficient to drive the high degree of hotspot divergence we observed. However, if the bottleneck strength was very high (*s* = 0.9) in both populations, elevated error rates in hotspot calling could result in a lack of hotspot overlap that mirrors the divergence we observed between populations. In this simulation, there was a similar percent of shared hotspots as observed in the actual populations (strong bottleneck: Lake Washington: 20.7%; Puget Sound: 19.8%; actual Puget Sound shared hotspots: 13.1%; actual Lake Washington shared hotspots: 19.1%). However, the strong bottleneck simulated was not realistic in comparison to the observed bottlenecks estimated by PSMC. Both Lake Washington and Puget Sound exhibited population size reductions that were moderate in comparison (Lake Washington had a 28.94% reduction in population size; Puget Sound had a 44.05% reduction in population size).

Based on the demographic histories we estimated, Lake Washington experienced a less intense bottleneck than Puget Sound. We therefore also used simulations to explore the expected hotspot overlap if only one of the populations experienced a strong bottleneck. If Puget Sound experienced a strong bottleneck and Lake Washington experienced a weak bottleneck, 36.7% of hotspots were shared in the simulated Lake Washington population and 20.5% of hotspots were shared in the simulated Puget Sound population (actual Lake Washington shared hotspots: 19.1%, actual Puget Sound shared hotspots: 13.1%). Except for a scenario where both populations underwent a severe bottleneck in the past, our simulations suggest that demographic history alone is not sufficient to completely explain the divergence we observed in hotspot location between populations.

### Nucleotide Diversity and Genetic Differentiation Differ between Hotspots and Coldspots

Selection and local recombination rate can affect nucleotide diversity within populations and genetic differentiation between populations (reviewed in Cutter and Payseur 2013). Nucleotide diversity tends to be reduced in regions of low recombination due to selection on linked sites, either through hitchhiking from selective sweeps (Maynard-Smith and Haigh 1974; Kaplan et al. 1989; Przeworski et al. 2005; Charlesworth et al. 2009) or from background selection (Charlesworth et al. 1993, 1997; Hudson and Kaplan 1995; Charlesworth 2012). Both forms of selection increase genetic differentiation between populations at linked sites (Charlesworth et al. 1997, 2009; Przeworski et al. 2005; Reed and Tishkoff 2006; Charlesworth 2012; Burri 2017; Comeron 2017). To determine if the low recombination regions (i.e., coldspots) we identified through our LD-based approach matched predicted patterns of nucleotide diversity and differentiation, we measured *π* and *F*_ST_ across the complete set of 2-kb coldspots. Coldspots in Puget Sound had significantly lower estimates of *π*, compared with the remainder of the genome (Puget Sound coldspots: 0.0015; genome-wide: 0.0024; *P* < 0.001; supplementary fig. 9, Supplementary Material online). Nucleotide diversity in Lake Washington coldspots was also significantly lower when compared with genome-wide estimates (Lake Washington coldspots: 0.0030; genome-wide: 0.0031; *P* = 0.011; supplementary fig. 9, Supplementary Material online). Both populations had significantly elevated levels of *F*_ST_ at coldspots compared with the remainder of the genome (Lake Washington coldspots *F*_ST_: 0.078; Lake Washington genome-wide *F*_ST_: 0.030; Puget Sound coldspots *F*_ST_: 0.058; Puget Sound genome-wide *F*_ST_: 0.030; *P* < 0.001; supplementary fig. 9, Supplementary Material online), matching theoretical predictions.

The effects of selection extend over a smaller distance in regions of the genome with high recombination rates. Consequently, nucleotide diversity is higher and genetic differentiation between populations is generally lower compared with regions with reduced recombination rates (Huang et al. 2005; Keinan and Reich 2010). True hotspots of recombination should therefore have low *F*_ST_ relative to coldspots. False hotspots signatures can occur at sites under strong positive selection (Reed and Tishkoff 2006). However, in these cases, sites closely linked to the locus under selection should have reduced nucleotide diversity and elevated genetic differentiation, similar to coldspots (Ptak et al. 2004; Reed and Tishkoff 2006; Charlesworth et al. 2009). We examined whether the population-specific and shared hotspots we detected in Lake Washington and Puget Sound exhibited signatures of true hotspots or whether many appeared to be false positives, driven by selective sweeps. In Lake Washington, estimates of *F*_ST_ were slightly elevated compared with the remainder of the genome (Lake Washington population-specific: 0.033, *P* < 0.001; Lake Washington shared: 0.032, *P* = 0.229; Lake Washington genome-wide 0.030; supplementary fig. 9, Supplementary Material online). However, inconsistent with a signature of positive selection, *π* was slightly elevated in population-specific and shared hotspot, compared with the rest of the genome (Lake Washington population-specific: 0.0032, *P* = 0.238; Lake Washington shared: 0.0035, *P* = 0.011; supplementary fig. 9, Supplementary Material online). The same pattern was present in Puget Sound. Shared and unique hotspots had slightly elevated *F*_ST_ compared with the remainder of the genome (Puget Sound population-specific: 0.041, *P* < 0.001; Puget Sound shared: 0.033, *P* = 0.183; Puget Sound genome-wide: 0.030; supplementary fig. 9, Supplementary Material online). But, *π* was also elevated, rather than reduced, at hotspots (Puget Sound population-specific: 0.0027, *P* < 0.001; Puget Sound shared: 0.0026, *P* = 0.104; genome-wide: 0.0024; supplementary fig. 9, Supplementary Material online).

### Hotspots Are Not Clustered in Specific Regions across Chromosomes

Given broad-scale recombination rates were higher toward the terminal chromosome ends in males, we examined whether hotspots were enriched in particular regions of chromosomes. We first examined if hotspots tended to occur in clusters throughout the genome. We found both shared and population-specific hotspots were mostly spaced at intervals >200 kb away from the nearest hotspot (Lake Washington population-specific: 315 kb; Puget Sound population-specific: 193 kb; supplementary fig. 14, Supplementary Material online). Shared hotspots were spaced at even greater intervals (average distance between shared hotspots: 1.35 Mb; supplementary fig. 14, Supplementary Material online), indicating both types of hotspots did not occur in clusters. At a broader scale, we also did not observe an enrichment of hotspots toward the terminal ends of chromosomes. Population-specific hotspots were more often located in the internal region (internal 50% of the chromosome) rather than the terminal ends of chromosomes (terminal 25% of the chromosome) (Lake Washington terminal hotspots: 38.4%; Lake Washington internal hotspots: 61.6%; Puget Sound terminal hotspots: 37.9%; Puget Sound internal hotspots: 62.1%; two proportion *Z*-test; *P* < 0.001). The same pattern was observed with hotspots shared between the two populations (terminal hotspots: 41.2%; internal hotspots: 58.5%; two proportion *Z*-test; *P* = 0.002). Lake Washington population-specific hotspots had 37.8% hotspots in the terminal ends of chromosomes and 62.2% hotspots were in the internal regions (two proportion *Z*-test; *P* < 0.001). Puget Sound had 33.9% hotspots near the terminal ends of chromosomes and 66.1% hotspots near the internal regions (two proportion *Z*-test; *P* < 0.001). Although there were fewer hotspots in the terminal regions of chromosomes, the hotspots that did occur in these regions were more intense, with higher rates of recombination than hotspots in internal regions of chromosomes (Lake Washington terminal regions: 0.206 *ρ*/bp; Lake Washington center: 0.074 *ρ*/bp; Puget Sound terminal regions: 0.399 *ρ*/bp; Puget Sound center: 0.252; Wilcoxon rank test; *P* < 0.001). Therefore, the high broad-scale recombination rates observed in the terminal ends of chromosomes may be driven by fewer hotspots that have greater intensities.

### Hotspots Are Enriched around TSSs

Hotspot localization in genomes varies among taxa. In yeast, birds, and some plants, where hotspots are evolutionarily conserved, hotspots tend to be enriched within TSSs (Pan et al. 2011; Tischfield and Keeney 2012; Auton et al. 2013; Singhal et al. 2015; Kawakami et al. 2017). In mammals with rapidly evolving hotspots, hotspots are typically located away from genic regions (Brick et al. 2012; Brunschwig et al. 2012). We investigated whether threespine stickleback fish hotspots mimic either of the patterns seen in other systems. We found an enrichment of hotspots around TSSs, compared with random permutations of hotspots (Lake Washington: 26% of hotspots fell within 3 kb of a TSS, *P* = 0.034; Puget Sound: 29% of hotspots fell within 3 kb of a TSS, *P* < 0.001; supplementary fig. 15, Supplementary Material online). This pattern also held when examining only population-specific hotspots (Lake Washington: *P* = 0.007; Puget Sound: *P* < 0.001; supplementary fig. 15, Supplementary Material online); however, shared hotspots were not enriched in TSSs compared with random permutations (Lake Washington: *P* = 0.370; Puget Sound: *P* = 0.827; supplementary fig. 15, Supplementary Material online). The lack of significant enrichment of shared hotspots around TSSs is likely due to the small sample size. When we randomly drew samples from the population-specific hotspots that were equal in size to the shared hotspot pools, there was no longer enrichment around TSSs (Lake Washington: *P* = 0.947; Puget Sound: *P* = 0.808).

We further investigated what types of genes were enriched around hotspots. We looked for enrichment of GO terms in the genes whose TSSs were within 3 kb of hotspots (86 TSSs were within 3 kb of shared hotspots, 465 TSSs were within 3 kb of Lake Washington population-specific hotspots, and 831 TSSs were within 3-kb Puget Sound population-specific hotspots) (supplementary table 4, Supplementary Material online). We found a significant enrichment of GO terms associated with these genes in each of the hotspot sets compared with random permutations of genes from the remainder of the genome (shared hotspots: 36 GO terms; supplementary table 5, Supplementary Material online; Puget Sound population-specific hotspots: 67 GO terms; supplementary table 6, Supplementary Material online; Lake Washington population-specific hotspots: 32 GO terms; supplementary table 7, Supplementary Material online; *P* < 0.001). Most of the GO terms were not shared among the three hotspot sets (supplementary table 8, Supplementary Material online), indicating hotspots are not localizing around a specific type of gene in each population. The unique GO terms for each hotspot set represent broad cellular functions, like enzymatic activity, regulation of transcription or signaling pathways.

### Hotspots Do Not Have GC-Biased Nucleotide Substitutions

Recombination leaves distinct signatures of nucleotide substitution across the genome (Duret and Arndt 2008; Webster and Hurst 2012; Mugal et al. 2015). Over time, the repair of heteroduplex DNA during meiosis favors the substitution of GC nucleotides over AT nucleotides, which increases the frequency of GC nucleotides, leading to GC-biased base composition (Marais 2003; Meunier and Duret 2004; Lesecque et al. 2013). Regions of the genome with higher recombination rates tend to have higher GC-biased base composition (Kong et al. 2002; Jensen-Seaman et al. 2004; Meunier and Duret 2004; Stevison and Noor 2010; Singhal et al. 2015; Kawakami et al. 2017). However, it is unclear whether this correlation is because more recombination leads to more GC-bias (Lesecque et al. 2013) or if regions with higher GC content are more likely to be targets of recombination (Meunier and Duret 2004; Fryxell and Moon 2005). To determine whether regions of higher recombination rate showed signatures of GC-biased gene conversion, we first calculated equilibrium GC content (1962; Meunier and Duret 2004; Singhal et al. 2015) in 2-kb nonoverlapping windows across the genome. Consistent with patterns of GC bias observed in other species, we observed a significant positive correlation between recombination rates in 2-kb windows and equilibrium GC content in both Lake Washington (Spearman’s rank correlation; rho = 0.076; *P* < 0.001) and Puget Sound (Spearman’s rank correlation; rho = 0.126; *P* < 0.001). However, when we looked at equilibrium GC content specifically within hotspots and coldspots across the genome, we did not find a significant difference in mean equilibrium GC compared with genome-wide means (table 1). If hotspots are more recently derived, they may not have had a strong impact on GC-biased nucleotide substitutions locally in the genome. The moderate positive correlation we observed in the genome-wide data likely reflects regions of the genome with historically high recombination rates that occur over broader scales. Hotspots would not be detected in these regions if the background rate is also elevated. These results are consistent with a model of recombination hotspots not being directly targeted to regions of the genome with ancestrally high GC content.

**Table 1:** Mean Equilibrium GC Content (±SE)

	Lake Washington	Puget Sound
Population-specific hotspots	0.418 (±0.0016)^a^	0.415 (±0.0012)^b^
Shared hotspots	0.419 (±0.0044)^a^	0.419 (±0.0036)^b,c^
Coldspots	0.416 (±0.0014)^a^	0.411 (±0.0012)^b,c^
Genome-wide	0.420 (±0.0003)^a^	0.417 (±0.0003)^b^

a–c Groups significantly different within populations by Wilcoxon rank test; *P* < 0.05; adjusted for multiple testing with a Bonferroni correction.

### Recombination Rates Are Elevated in the PAR and Reduced across the Remainder of the X Chromosome

In threespine stickleback, crossing over between the X and Y chromosomes is restricted to a ∼2.5-Mb pseudoautosomal region (PAR) (Peichel et al. 2004; Roesti et al. 2013; Yoshida et al. 2014; White et al. 2015). Because of the potential for high rates of crossing over in the PAR, we estimated population-scaled recombination rates for this region independently from the autosomes. The average recombination rate in the PAR was 0.232 *ρ*/bp for Puget Sound and 0.129 *ρ*/bp for Lake Washington. These rates were significantly higher than the average recombination rate across the autosomes (Wilcoxon rank test; Lake Washington autosome average rate: 0.035 *ρ*/bp, *P* < 0.001; Puget Sound autosome average rate: 0.072 *ρ*/bp, *P* < 0.001; supplementary fig. 16*A*, Supplementary Material online). Although we observed some fine-scale variation in recombination rates across the PAR (supplementary fig. 16*A*, Supplementary Material online), we identified very few hotspots, which may be due to the increased background recombination rate across the region.

We also estimated population-scaled recombination rates across the entire X chromosome using only females. The average recombination rate across the X chromosome (including the PAR) was 0.052 *ρ*/bp for Lake Washington and 0.103 *ρ*/bp for Puget Sound. With the PAR excluded, the average recombination rate was 0.024 *ρ*/bp and 0.052 *ρ*/bp for Lake Washington and Puget Sound, respectively (supplementary fig. 16*B*, Supplementary Material online). We tested whether the reduced recombination rate on the X chromosome, relative to the autosomes (Lake Washington average autosomal rate: 0.035; Puget Sound average autosomal rate: 0.072), was due to the smaller sample size used for the X chromosome. We downsampled a single autosome (chromosome one), matching the sample size for the X chromosome rate estimation, to test if there was an effect on recombination rate. After downsampling, we did not observe a significant change in average recombination rate for chromosome one (Wilcoxon one-sample test; Lake Washington: *P* = 0.073; Puget Sound: *P* = 0.156; supplementary table 9, Supplementary Material online), indicating the reduced rate across the X chromosome is likely not an effect of sample size and is instead an intrinsic feature of the demographic history of the threespine stickleback sex chromosomes.

### PRDM Genes Are Weakly Associated with Threespine Stickleback Recombination Hotspots

Hotspots in many species are targeted to specific regions of the genome by DNA-binding motifs (Kon et al. 1997; Steiner et al. 2002; Myers et al. 2008; Baudat et al. 2010). In species where PRDM9 targets recombination hotspots to specific regions of the genome, the zinc finger domain of PRDM9 is typically under strong positive selection (Oliver et al. 2009; Myers et al. 2010) and the protein contains functional KRAB and SSXRD domains (Baker et al. 2017). Recent work has suggested that the KRAB domain is important for recruiting other recombination proteins to where PRDM9 is bound (Grey et al. 2017; Imai et al. 2017). In teleost fish, two paralogs of *Prdm9* have been identified, *Prdm9α* which contains all the protein domains and *Prdm9β* which lacks the KRAB and SSXRD domains (Baker et al. 2017). Threespine stickleback fish appear to have lost *Prdm9α* but retain *Prdm9β*. The function of *Prdm9β* is unknown. Consistent with a lack of function directing recombination hotspots, we did not observe strong signatures of positive selection in the zinc finger domain of *Prdm9β*. We found zero fixed differences between threespine and blackspotted stickleback within *Prdm9β.* There was one synonymous and one nonsynonymous mutation at moderate frequency in Lake Washington and two synonymous and three nonsynonymous mutations at moderate frequency in Puget Sound, indicating these mutations are likely not causing the population-specific localization of hotspots we observed between Lake Washington and Puget Sound.

We also examined whether the predicted binding sites of any of the 11 previously annotated *Prdm* genes in threespine stickleback fish were enriched in recombination hotspots. Less than 14% of hotspots contained any of the predicted PRDM zinc finger binding domain motifs (supplementary table 10, Supplementary Material online). However, six of the motifs were significantly enriched in hotspots, including PRDM9β, when compared with scrambled motifs of the same size and GC content (supplementary table 10, Supplementary Material online), indicating *Prdm* genes could have some role in localizing a subset of recombination hotspots. Outside of PRDM9 in mammals, multiple DNA-binding motifs assist with hotspot targeting in other systems such as *S. pombe* (Kon et al. 1997; Steiner et al. 2002, 2009). To see if other DNA motifs were targeting hotspots in threespine stickleback fish, we searched for motifs enriched in hotspots. The most significant motifs identified were simple mono- or di-nucleotide repeats which were present only in a subset of the hotspots (supplementary fig. 17, Supplementary Material online). These repeats were not specific to hotspots as they were also found in GC-matched coldspots.

## Discussion

### Broad-Scale Recombination Rates Are Conserved among Threespine Stickleback Populations

At a broad scale, recombination rates across the threespine stickleback genome were conserved between the two populations. This broad-scale conservation of recombination rates is a feature observed in many taxa (Kong et al. 2002; Serre et al. 2005; Fledel-Alon et al. 2009; Stevison et al. 2016) and may reflect the necessity of crossing over for the proper segregation of chromosomes during meiosis (Mather 1936; Kaback et al. 1992; Davis and Smith 2001). Additionally, we observed differential rates of recombination associated with broad genomic regions that have been observed in other systems. For one, we observed higher recombination rates toward the terminal chromosome ends. In many species, the terminal chromosome ends have higher rates of recombination (Kong et al. 2002; Barton et al. 2008; Roesti et al. 2013; Berner and Roesti 2017; Haenel et al. 2018; Sardell et al. 2018), which is thought to be driven by male-specific localization of recombination (Broman et al. 1998; Singer et al. 2001; Moen et al. 2008). Our LD-based method estimates sex-averaged recombination rates, which does not allow us to test whether the pattern we observed around the terminal chromosome ends is driven by males. However, sex-specific genetic linkage maps between the Japan Sea stickleback (*Gasterosteus nipponicus*) and the threespine stickleback (*G. aculeatus*) corroborate this pattern (Sardell et al. 2018). The mechanisms that drive this pattern remain unclear (Hurst et al. 2005; Stapley et al. 2017; Haenel et al. 2018). We showed that hotspots are not clustered near the terminal chromosome ends, which indicates an overall increase in hotspot number is likely not responsible. However, the hotspots that we did detect in the terminal chromosome ends were more intense, with higher recombination rates overall. Therefore, one possible mechanism to increase recombination rates may be to increase intensity of existing hotspots in the terminal chromosome ends, rather than increase the total hotspot number.

Across the X chromosome, we observed distinct regional differences in recombination rates. Within the PAR, we observed higher recombination rates compared with autosomes. Recombination rates in PARs are often orders of magnitude above autosome-wide averages, as an obligate crossover should occur between the X and Y chromosomes in these small regions during every male meiosis (Otto et al. 2011; Kauppi et al. 2012; Hinch et al. 2014). Outside of the PAR, we found there was a lower average recombination rate on the X chromosome compared with autosomes. In populations with an equal sex ratio, recombination only occurs on the X chromosome in two-thirds of meioses each generation (Schaffner 2004). This results in a recombination rate across the X chromosome that is two-thirds of the genome-wide average (Kong et al. 2002). Consistent with this theoretical prediction, the X chromosome recombination rates we estimated with our LD-based approach are also approximately two-thirds of the average autosome recombination rates (Lake Washington: 0.686 of autosomes; Puget Sound: 0.722 of autosomes).

Overall, the genome-wide average recombination rate for Puget Sound was 2-fold higher than in Lake Washington. Rate variation between populations or species can be driven by several processes. Structural variation (i.e., inversions, chromosomal rearrangements, and copy number variants) can contribute to rate variation among genomes. Indeed, recombination rates have been shown to vary across chromosomal regions due to segregating inversions between marine and freshwater populations of threespine stickleback (Jones, Grabherr, et al. 2012; Glazer et al. 2015). However, structural variation would only explain the genome-wide recombination differences we observed if genomic rearrangements were heterozygous within a single population. In threespine stickleback fish, most structural variants are fixed between marine and freshwater populations though there are a few exceptions (Jones, Grabherr, et al. 2012; Chain et al. 2014; Hirase et al. 2014; Roesti et al. 2015). Over longer evolutionary timescales, recombination rate also can evolve neutrally (Dumont and Payseur 2008), driving genome-wide rate variation between species. However, neutral divergence is likely not occurring at a pace that would alter genome-wide recombination rates between recently diverged populations of threespine stickleback fish. One plausible explanation for the observed rate differences is differences in demographic history between the Lake Washington and Puget Sound populations. A larger effective population size could increase the population-scaled recombination rate (Burt 2000; Charlesworth 2009; Adrian et al. 2016). In threespine stickleback, marine populations typically have a larger *N*_e_ than freshwater populations (Gow et al. 2006; Makinen et al. 2006; DeFaveri and Merila 2015), consistent with our observed pattern of a higher recombination rate in Puget Sound relative to Lake Washington.

### Demographic Processes Do Not Completely account for Hotspot Divergence

LD-based estimates of recombination rates can be affected by demographic processes that change patterns of LD across the genome (Chan et al. 2012; Johnston and Cutler 2012; Wall and Stevison 2016; Dapper and Payseur 2018). The duration and timing of these events can have varying effects on hotspot identification, often reducing the power to detect hotspots and increasing the rate of errors (Dapper and Payseur 2018). Threespine stickleback fish have a complex history of bottleneck events and population expansions over the last 10–15 thousand years which vary across geographic regions (Bell and Foster 1994) and our analyses have shown that Puget Sound and Lake Washington are distinct populations. Consistent with what has been observed in other populations, we found Puget Sound and Lake Washington both experienced past bottleneck events. Based on simulations, demographic history could have some role in the observed divergence in hotspot location between Lake Washington and Puget Sound populations, but it seems likely that population demography does not completely explain the pattern. Only in the scenario where both populations experienced a strong bottleneck do error rates rise high enough to mimic the observed divergence in hotspot location. This bottleneck strength exceeded the reductions in population size we estimated through PSMC.

Our estimates of effective population size over time revealed that Lake Washington and Puget Sound did not experience similar fluctuations. Both populations began with effective population sizes that largely parallel those observed in other threespine stickleback fish populations (Liu and Hansen 2017; Ravinet et al. 2018). Puget Sound then experienced a larger population expansion roughly 18,000 years ago, followed with a decrease in population size at ∼8,000 years ago. Lake Washington had a slight increase in population size, followed by a small bottleneck around the same time, but overall changes in effective population size were more stable in this population. Little is known about the colonization history of these specific populations, making it unclear why Puget Sound experienced a stronger bottleneck over time.

### Recombination Hotspots Are Not Artifacts of Selection

Positive selection can alter local patterns of LD, which can mimic the signal of a recombination hotspot (Przeworski et al. 2005; Reed and Tishkoff 2006). In these cases, nucleotide diversity at linked sites should be depleted as the selected variant is swept toward fixation (Kaplan et al. 1989; Charlesworth et al. 2009) and genetic differentiation between populations should increase (Charlesworth et al. 1997; Przeworski et al. 2005; Charlesworth et al. 2009; Charlesworth 2012; Comeron 2017). Some hotspots in Puget Sound and Lake Washington had elevated *F*_ST_ relative to background genomic levels. However, hotspots in both populations did not exhibit reduced nucleotide diversity compared with the genome-wide background, which is not consistent with strong positive selection. In the case of very strong positive selection, the selective sweep could resemble a recombination coldspot. Although we cannot discount that some of the hotspots or coldspots we identified are artifacts of selection, it seems unlikely that a majority of these are false positives. Genome-wide scans of selection between marine and freshwater populations of threespine stickleback fish have found a much smaller number of loci under strong positive selection (Hohenlohe et al. 2010; Jones, Chan, et al. 2012; Jones, Grabherr, et al. 2012). Balancing selection may also create a false positive hotspot signal, locally increasing nucleotide diversity, while reducing genetic divergence. Although this pattern matches the signal of a true hotspot, it is unlikely balancing selection is responsible for the entire set of population-specific hotspots in Lake Washington and Puget Sound. Even fewer loci with signatures of balancing selection have been identified between marine and freshwater populations (Hohenlohe et al. 2010).

### Recombination Hotspots Are Weakly Conserved between Marine and Freshwater Populations of Threespine Stickleback Fish

Of the 3,995 hotspots between Lake Washington and Puget Sound, only ∼15% of hotspots are shared, indicating many of the hotspots are recently derived within populations of threespine stickleback fish. One possible model for the observed divergence is that recombination hotspots are being directed by a PRDM9-dependent mechanism as observed in mammals. In mammals, strong positive selection acting on the zinc finger binding domain of PRMD9 has led to multiple distinct DNA-binding motifs between closely related species (Baudat et al. 2010; Myers et al. 2010; Pratto et al. 2014). This rapidly shifts the locations of hotspots (Brick et al. 2012; Pratto et al. 2014; Baker et al. 2015; Smagulova et al. 2016; Stevison et al. 2016). Typically, ∼40% of hotspots will contain a PRDM9 motif in mouse and humans (Myers et al. 2008; Baudat et al. 2010). In threespine stickleback fish, we found that <14% of hotspots had any PRDM motifs (PRDM9 motifs were only present in 190 Lake Washington hotspots and 260 Puget Sound hotspots), contrary to what we would expect if PRDM9 was controlling hotspot location in threespine stickleback. A lack of PRDM9 enrichment was also found among recombination hotspots in a genetic cross between populations of threespine stickleback (Sardell et al. 2018). In this study, a much smaller pool of hotspots was detected, limiting the overall power to detect enrichment. Because we observed a slight enrichment of PRDM motifs, we cannot completely discount some role of a PRDM protein in the regulation of recombination in threespine stickleback fish. *Prdm* genes in general have been shown to be involved with modifying histones which can affect chromatin structure (Fog et al. 2012; Vervoort et al. 2016). Additional work is necessary to explore the function of *Prdm* genes during meiosis in threespine stickleback fish. We did not observe an enrichment of any other DNA motifs in hotspots that would indicate a role of an alternative DNA-binding protein in localizing hotspots. Using a more direct approach to detect all possible locations where DSBs are occurring across the genome, such as ChIP-seq against DMC1 (Smagulova et al. 2011, 2016), may provide additional insights into what genomic features are targeting recombination hotspots in threespine stickleback fish.

Another possible model is that recombination hotspots can shift over short evolutionary timescales among regions of the genome that are susceptible to homologous recombination, such as regions of accessible chromatin. Both evolutionarily conserved and rapidly evolving hotspots tend to locate to regions of accessible chromatin (Pan et al. 2011; Comeron et al. 2012; Tischfield and Keeney 2012) or regions with histone 3 lysine 4 trimethylation (H3K4me3) (Smagulova et al. 2011; Brick et al. 2012; Tischfield and Keeney 2012; Baker et al. 2015; Powers et al. 2016; Marand et al. 2017). In taxa where hotspots are evolutionarily conserved, hotspots are highly enriched around TSSs (Pan et al. 2011; Tischfield and Keeney 2012; Auton et al. 2013; Singhal et al. 2015; Kawakami et al. 2017).This pattern could be due to either higher selective constraints at TSSs or the chromatin structure at TSSs. TSSs are often under purifying selection and if a genomic feature, like a DNA motif, is targeting hotspots to these regions, these features would also be preserved through purifying selection, maintaining the location of the hotspot (Tsai et al. 2010; Lam and Keeney 2015; Singhal et al. 2015; Kawakami et al. 2017). On the other hand, an open chromatin conformation could be driving this pattern. TSSs and the surrounding regions must be accessible for transcription to occur while also providing sites for Spo11 to bind, initiating recombination as Spo11 will create DSBs at any sites with accessible chromatin (Romanienko and Camerini-Otero 1999; Celerin et al. 2000; Pan et al. 2011). In Lake Washington and Puget Sound populations, we found some enrichment of hotspots at TSSs (Lake Washington: 26% of hotspots fell within 3 kb of a TSS; Puget Sound: 29% of hotspots fell within 3 kb of a TSS) which is similar to hotspot enrichment around TSS in taxa that do not have a functional PRDM9 protein. In birds and dogs, for example, ∼20–30% of hotspots overlap with TSSs (Auton et al. 2013; Singhal et al. 2015; Kawakami et al. 2017). Additional characterization is needed to determine if hotspots in threespine stickleback are occurring in regions of the genome that are already open due to transcription or if there is a mechanism that creates accessible chromatin specifically for DSB formation, like what is believed to occur with PRDM9 in mammalian species (Hayashi et al. 2005; Powers et al. 2016; Diagouraga et al. 2018).

## Supplementary Material

Supplementary data are available at *Genome Biology and Evolution* online.

## Supplementary Material

Supplementary_Material_evz090

## Notes

**Data deposition:** This project has been deposited at the NCBI Short Read Archive under the accession SRP137809. (https://www.ncbi.nlm.nih.gov/sra/SU3748706/overview; last accessed April 29, 2019 - to be released April 30, 2019.)
