# Supplementary material for: Divergent Fine-Scale Recombination Landscapes between a Freshwater and Marine Population of Threespine Stickleback Fish
Source: Genome Biol Evol. 2019 Apr 27;11(6):1552–72. doi: 10.1093/gbe/evz090 (PMC6553505; doi:10.1093/gbe/evz090)
Supplement: Supplementary_Material_evz090 [file supplementary_material_evz090.zip › SuppFigs_final_GBE_revision_submission.docx]

Supplemental Figure 1. Lake Washington and Puget Sound are genetically distinct populations. Population structure was estimated using FastStructure. The model with the best fit was a two- population model, separating Lake Washington (blue) from Puget Sound (red).


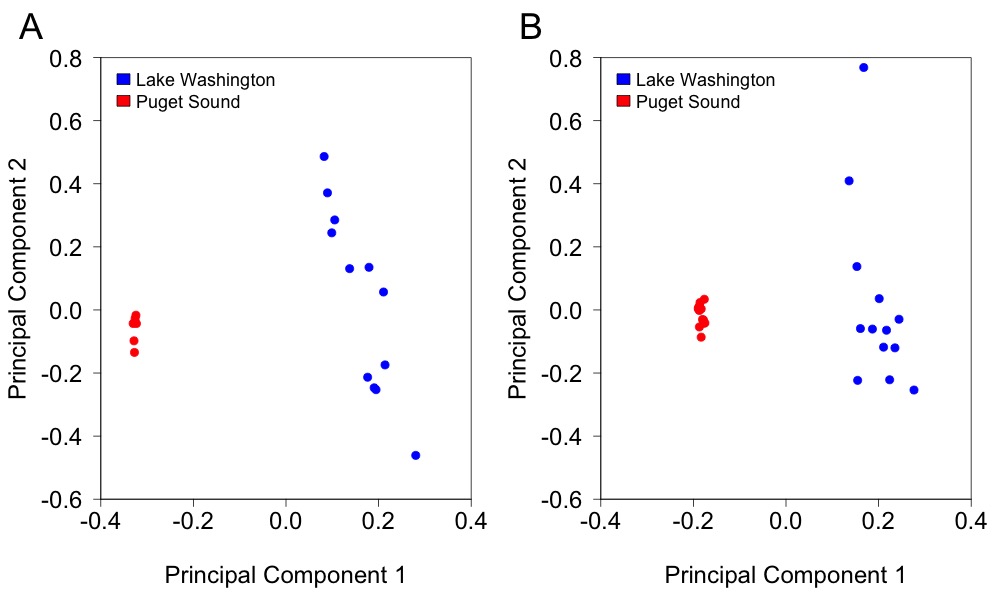


Supplemental Figure 2. Lake Washington and Puget Sound cluster by population. Principal component analysis was performed with individuals split by sex. Both (A) males and (B) females clustered by population (Lake Washington: blue; Puget Sound: red).

Supplemental Figure 3. Demographic history predictions for all individuals in Lake Washington and Puget Sound. PSMC was used to model the demographic history of (A) Lake Washington and (B) Puget Sound for all individuals from each population. The dark red lines in each plot refers to the predicted history of a single individual while the faded red lines represent 100 bootstrap predictions per each individual. The max current effective population size was restricted to 400,000 individuals.


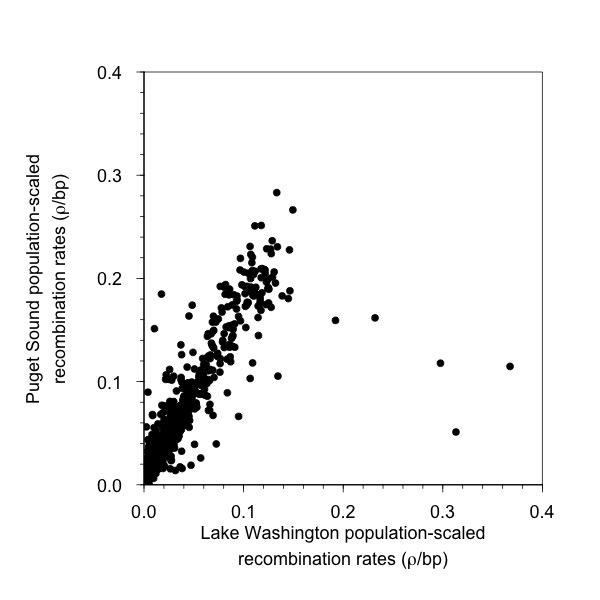


Supplemental Figure 4. Recombination rates are highly correlated between Lake Washington and Puget Sound. LD-based recombination rates were estimated using LDHelmet for every pair of adjacent SNPs. There is a significantly positive correlation between Lake Washington and Puget Sound LD-based recombination rates, averaged across 500 kb non-overlapping blocks (Spearman’s rank correlation; r = 0.931; p < 0.001).


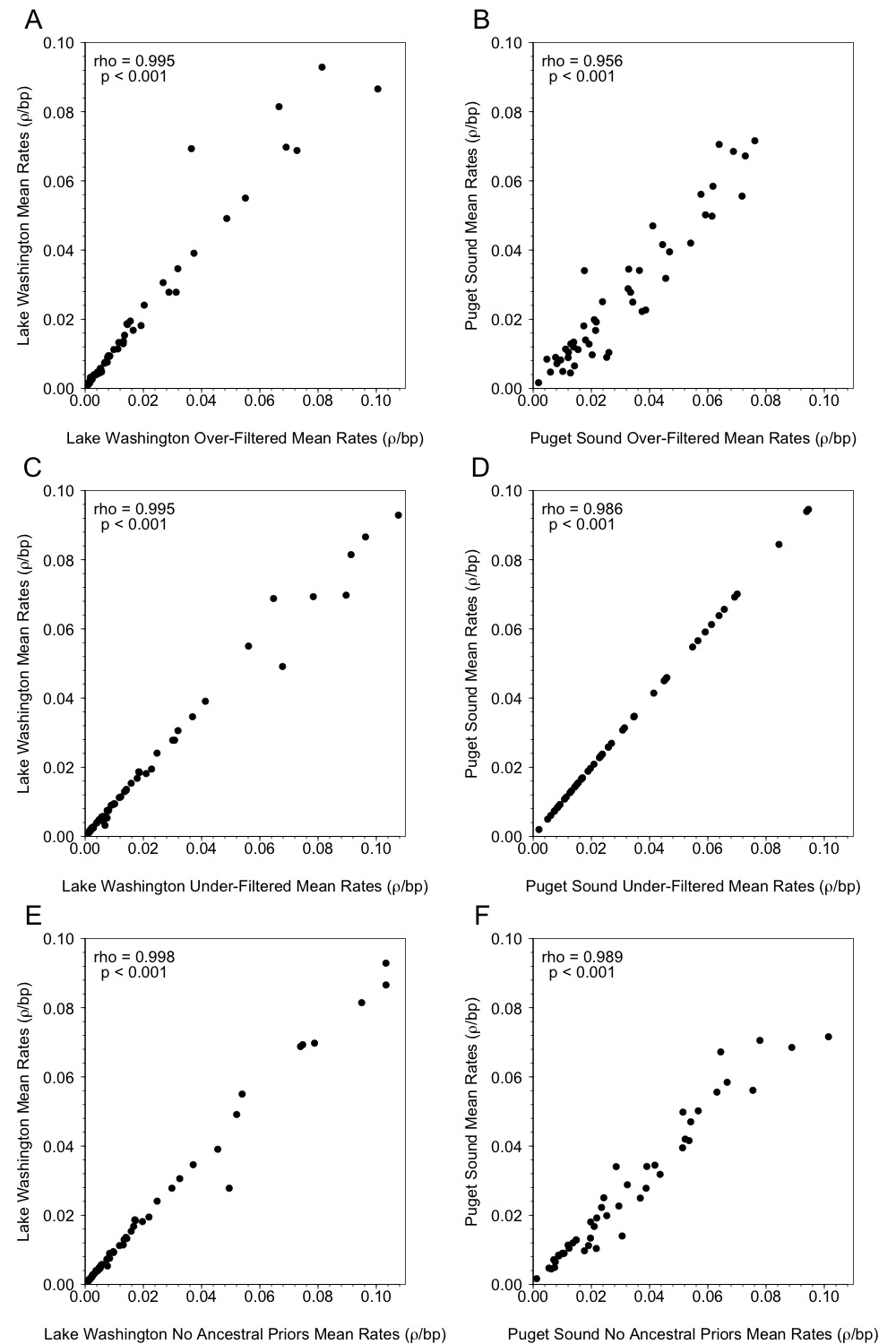


Supplemental Figure 5. Recombination rates are highly correlated between various filtering schemes and observed recombination rates. LD-based recombination rates were estimated using LDHelmet for every pair of adjacent SNPs. Observed recombination rates were compared to recombination rates estimated with (A and B) no ancestral state priors, (C and D) over-filtered SNP set, and (E and F) under-filtered SNP set. There is a significant positive correlation between all filtering schemes and the observed recombination rates (Spearman’s rank correlation; p < 0.001).


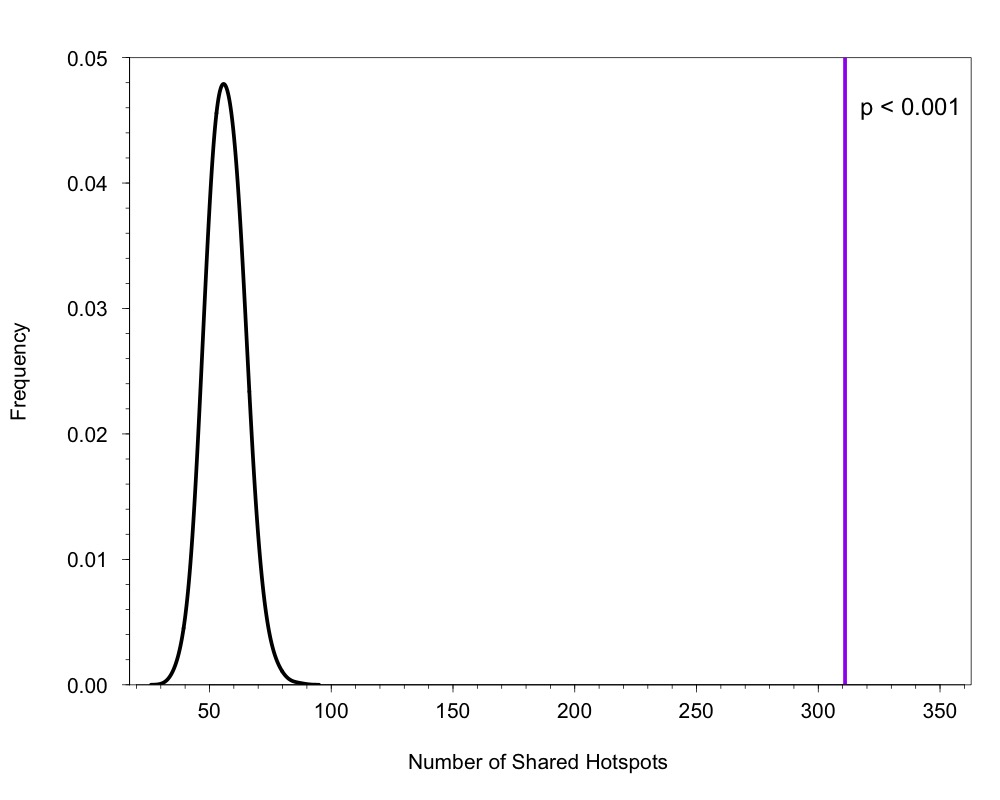


Supplemental Figure 8. Shared hotspots co-localize between Lake Washington and Puget Sound more often than expected by chance. 10,000 randomly drawn sets of hotspots had fewer overlapping hotspots (black) than the shared hotspots found through LDHelmet (purple). Hotspots were defined as shared between random populations if the midpoint of each hotspot fell within 3 kb of each other.

Supplemental Figure 9. Hotspots do not exhibit patterns consistent with strong positive selection. Nucleotide diversity and population divergence were calculated for both hotspots and coldspots. (A) Lake Washington coldspots had significantly lower π than the genome wide values, while shared hotspots had elevated π compared to the genome-wide values. (B) Lake Washington coldspots and population-specific hotspots had significantly elevated F_ST_ compared to genome-wide values. (C) Puget Sound coldspots had a decreased π compared to genome-wide values, while Puget Sound population-specific hotspots showed elevated π. (D) coldspots and population-specific hotspots had significantly elevated F_ST_ compared to genome-wide values.


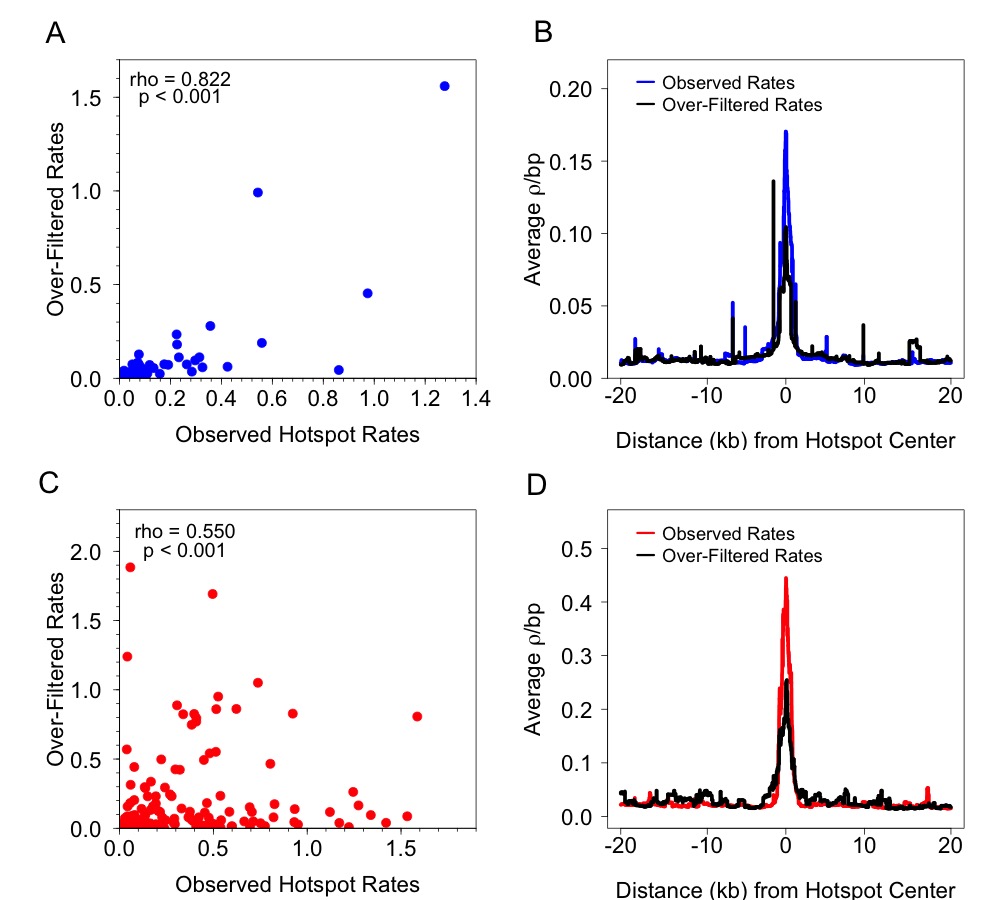


Supplemental Figure 10. Recombination rates are similar between the observed data set and the over-filtered SNP set. Hotspot rates were significantly positively correlated at observed hotspots in the observed rates and over-filtered rates in (A) Lake Washington and (C) Puget Sound. Mean recombination rates are shown across a 40 kb interval, flanking the center of observed hotspots in the observed recombination background and the same loci in over-filtered background for (B) Lake Washington and (D) Puget Sound.


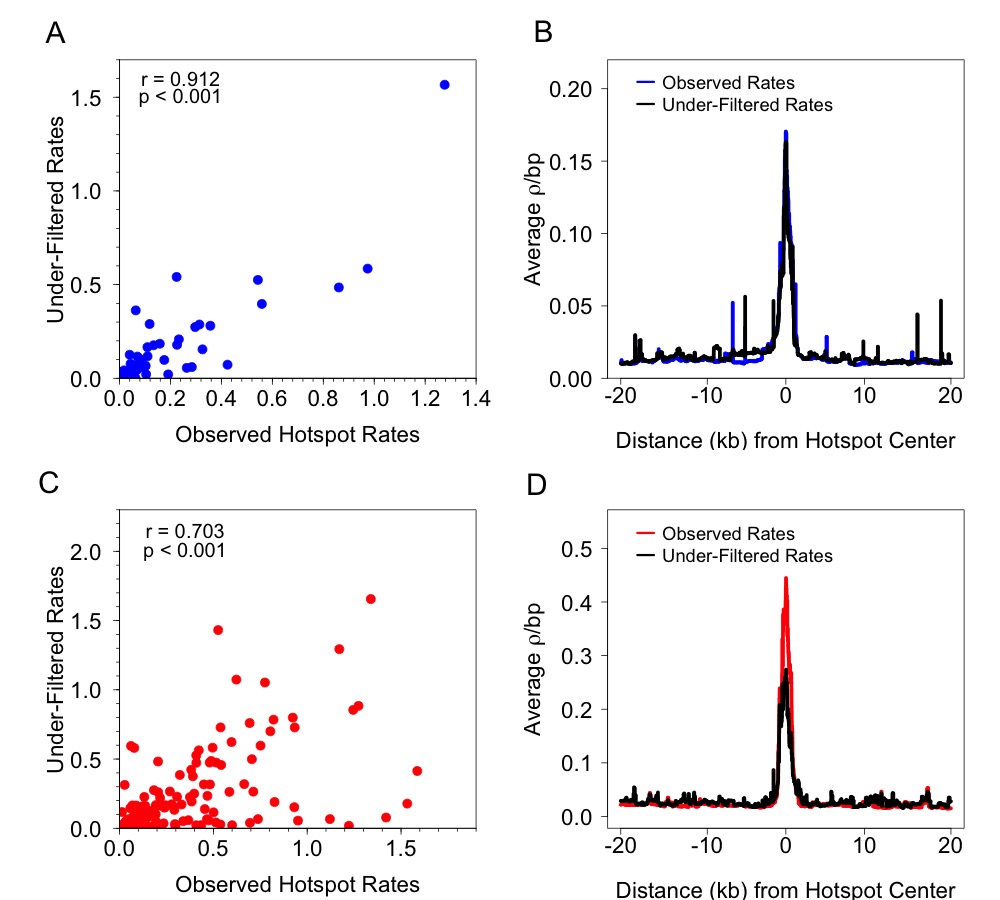


Supplemental Figure 11. Recombination rates are similar between the observed data set and the under-filtered SNP set. Hotspot rates were significantly positively correlated at observed hotspots in the observed rates and under-filtered rates in (A) Lake Washington and (C) Puget Sound. Mean recombination rates are shown across a 40 kb interval, flanking the center of observed hotspots in the observed recombination background and the same loci in the under-filtered background for (B) Lake Washington and (D) Puget Sound.


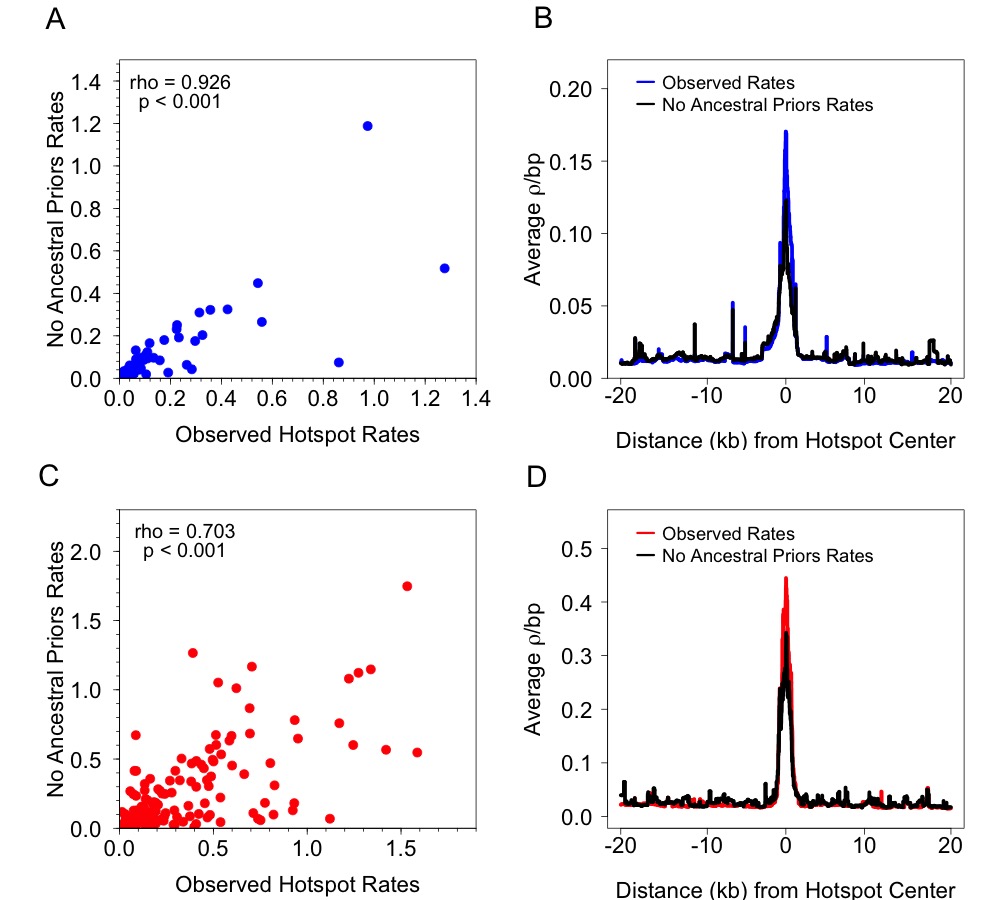


Supplemental Figure 12. Recombination rates are similar between the observed data set and without the ancestral state priors. Hotspot rates were significantly positively correlated at observed hotspots in the observed rates and no ancestral state priors rates in (A) Lake Washington and (C) Puget Sound. Mean recombination rates are shown across a 40 kb interval, flanking the center of observed hotspots in the observed recombination background and the same loci in the no ancestral states background for (B) Lake Washington and (D) Puget Sound.


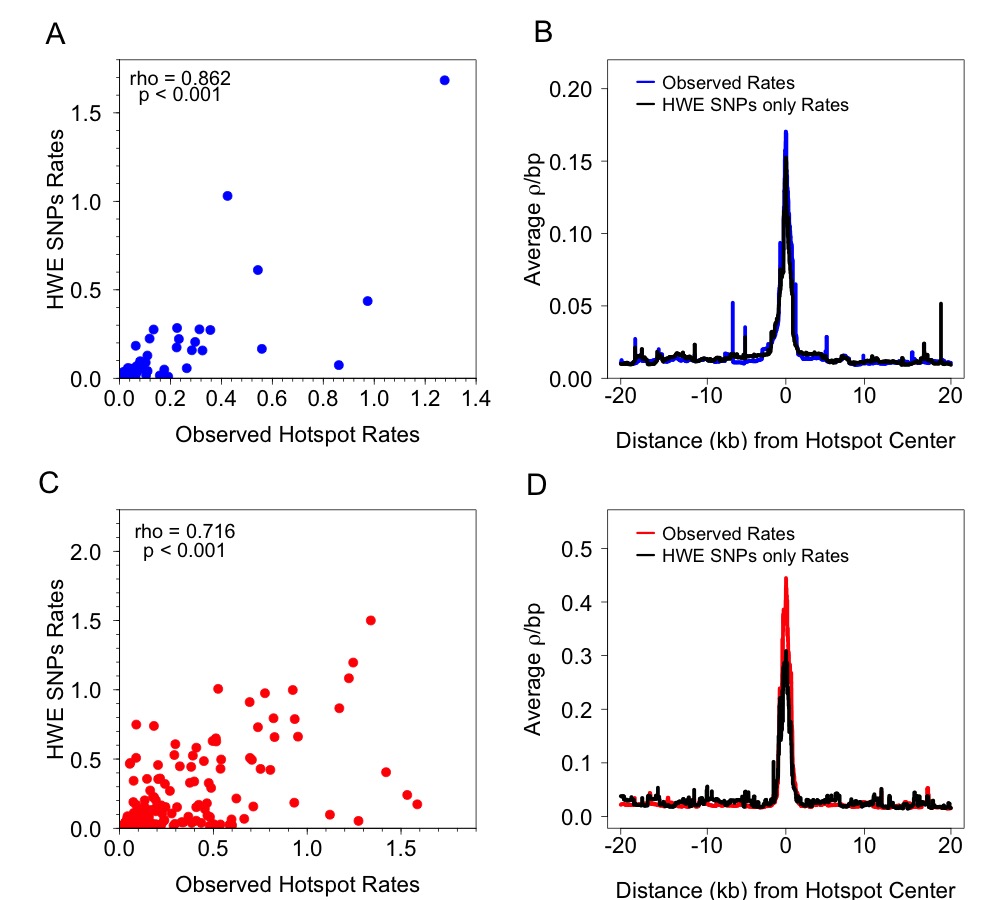


Supplemental Figure 13. Recombination rates are similar between the observed data set and with non-HWE SNPs excluded. Hotspot rates were significantly positively correlated at observed hotspots in the observed rates and HWE SNPs only rates in (A) Lake Washington and (C) Puget Sound. Mean recombination rates are shown across a 40 kb interval, flanking the center of observed hotspots in the observed recombination background and the same loci in the HWE SNPs only background for (B) Lake Washington and (D) Puget Sound.


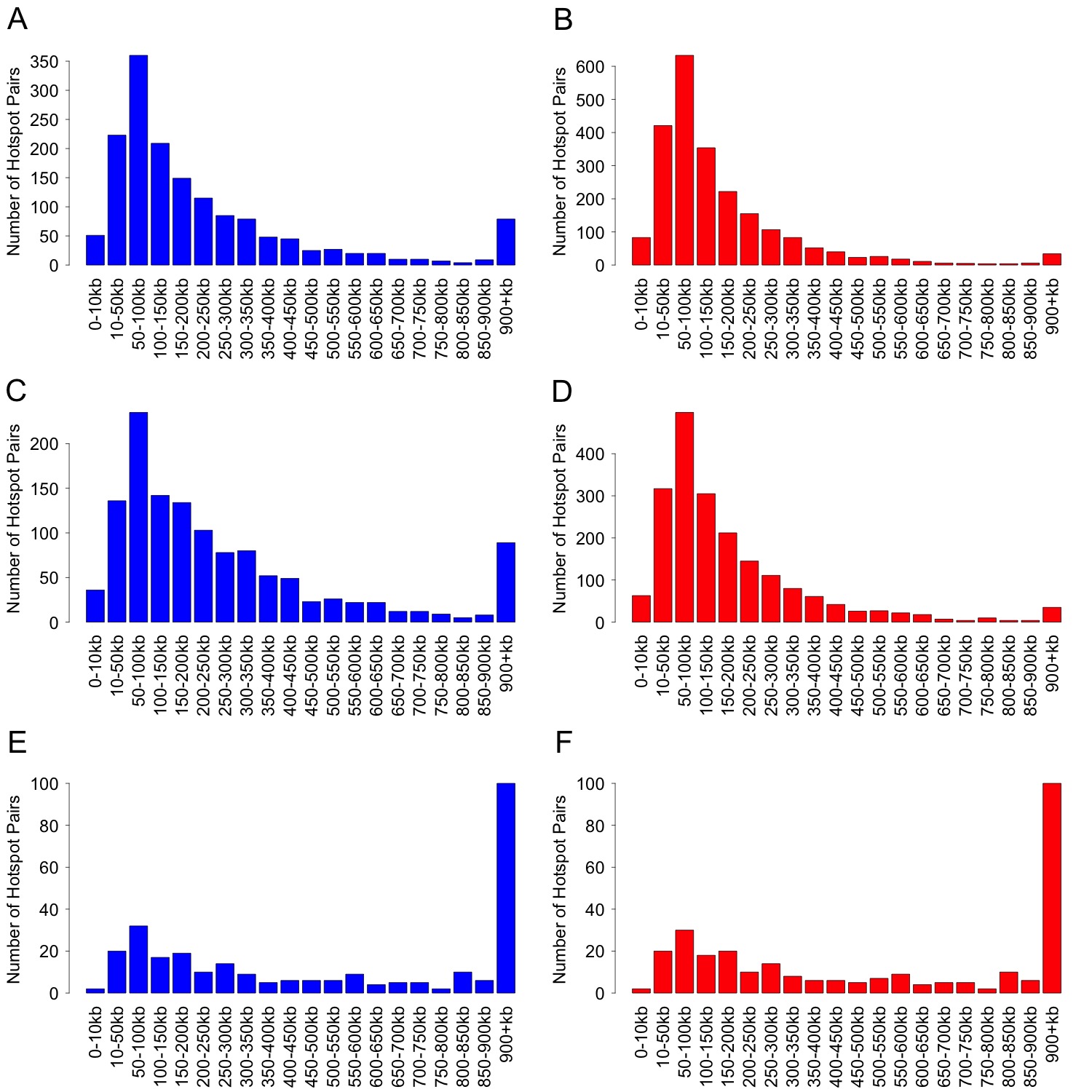


Supplemental Figure 14. Hotspots are mostly greater than 50 kb apart. The distance between hotspots was calculated and binned based on similar distances for (A and B) all hotspots, for (C and D) population-specific hotspots, and (E and F) shared hotspots. Lake Washington is shown in blue and Puget Sound in red.


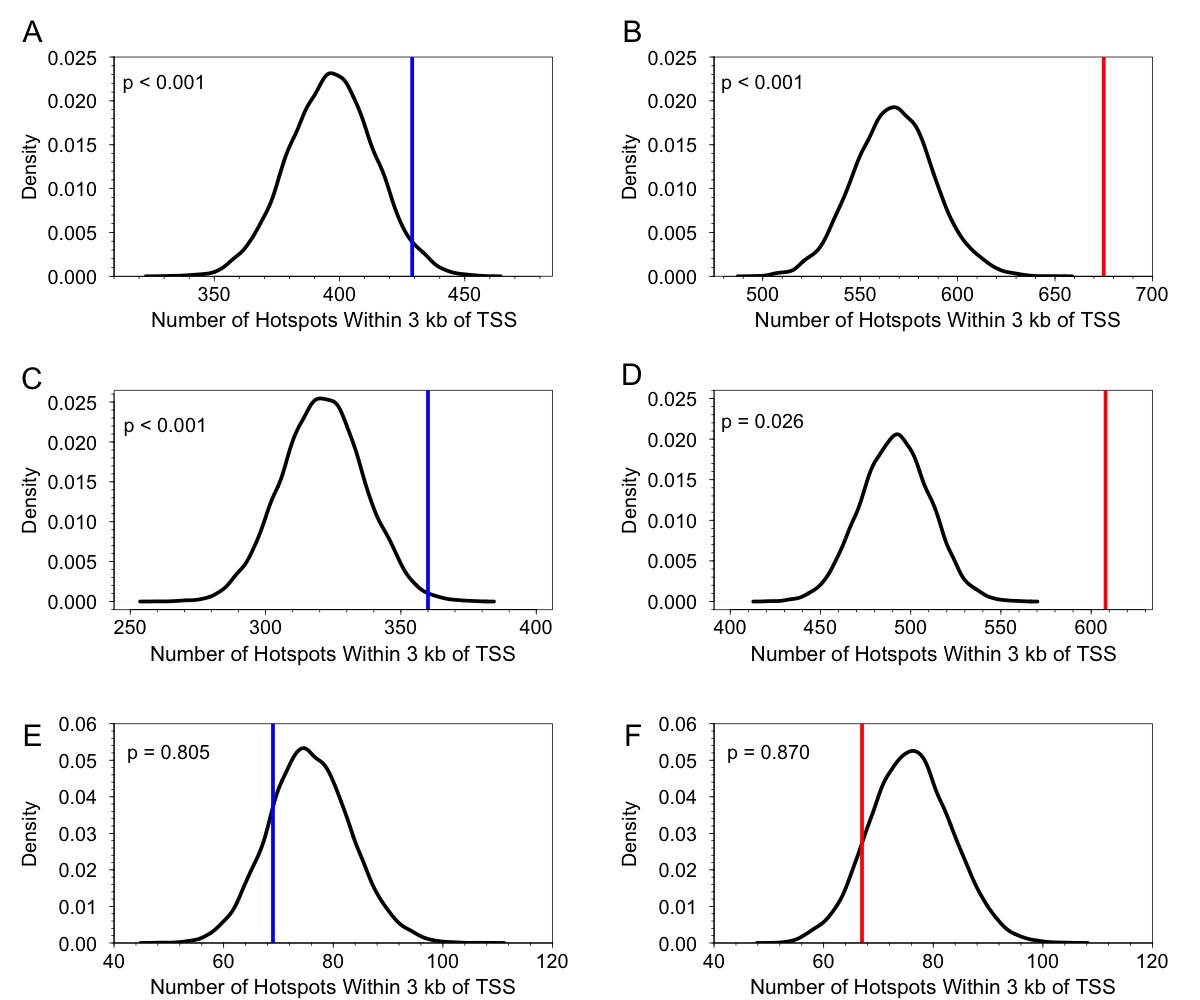


Supplemental Figure 15. Hotspots are enriched around transcription start sites. When the (A and B) total set of hotspots and (C and D) population-specific hotspots were considered, hotspots fell within 3 kb of a transcription start site more often than randomly drawn regions of the genome (black). This pattern was not observed with (E and F) shared hotspots, but this was likely due to the small number of these hotspots. Lake Washington is shown in blue and Puget Sound is shown in red.


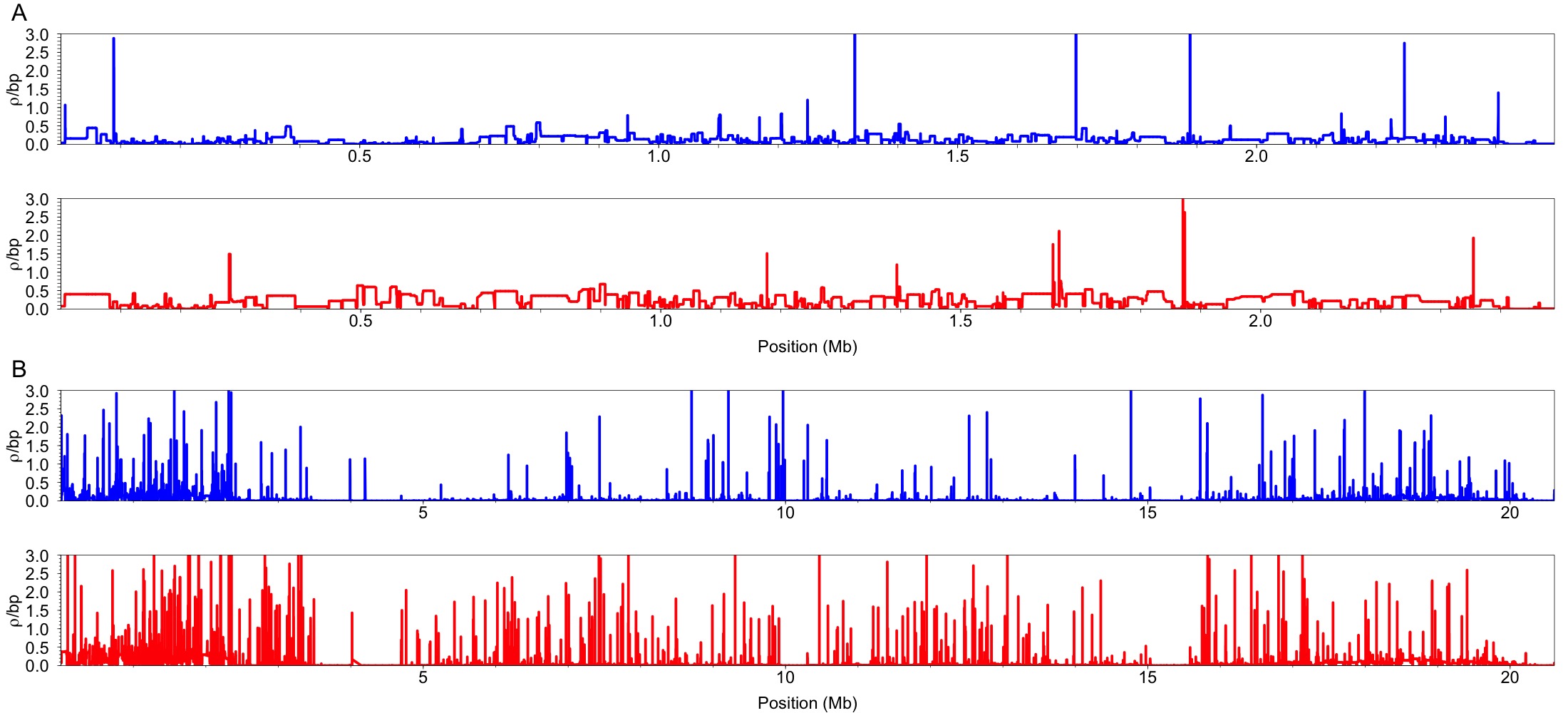


Supplemental Figure 16. Recombination rates across the X chromosome (chromosome 19). (A) LD-based recombination rates across the pseudoautosomal region (PAR). Overall, recombination rates are higher across the PAR than the autosomes. The PAR is the first ~2.5 Mb of linkage group 19. (B) LD-based recombination rates across the remainder of the X chromosome were lower than the PAR. Lake Washington is shown in blue and Puget Sound is shown in red.

Supplemental Figure 17. Simple repeat DNA motifs are enriched in recombination hotspots. Similar motifs were identified for every set of hotspots examined. Most motifs were simple mono- or di-nucleotide repeats.
