# Supplementary figures and images for: Divergent Fine-Scale Recombination Landscapes between a Freshwater and Marine Population of Threespine Stickleback Fish
Source: Genome Biol Evol. 2019 Apr 27;11(6):1552–72. doi: 10.1093/gbe/evz090 (PMC6553505; doi:10.1093/gbe/evz090)

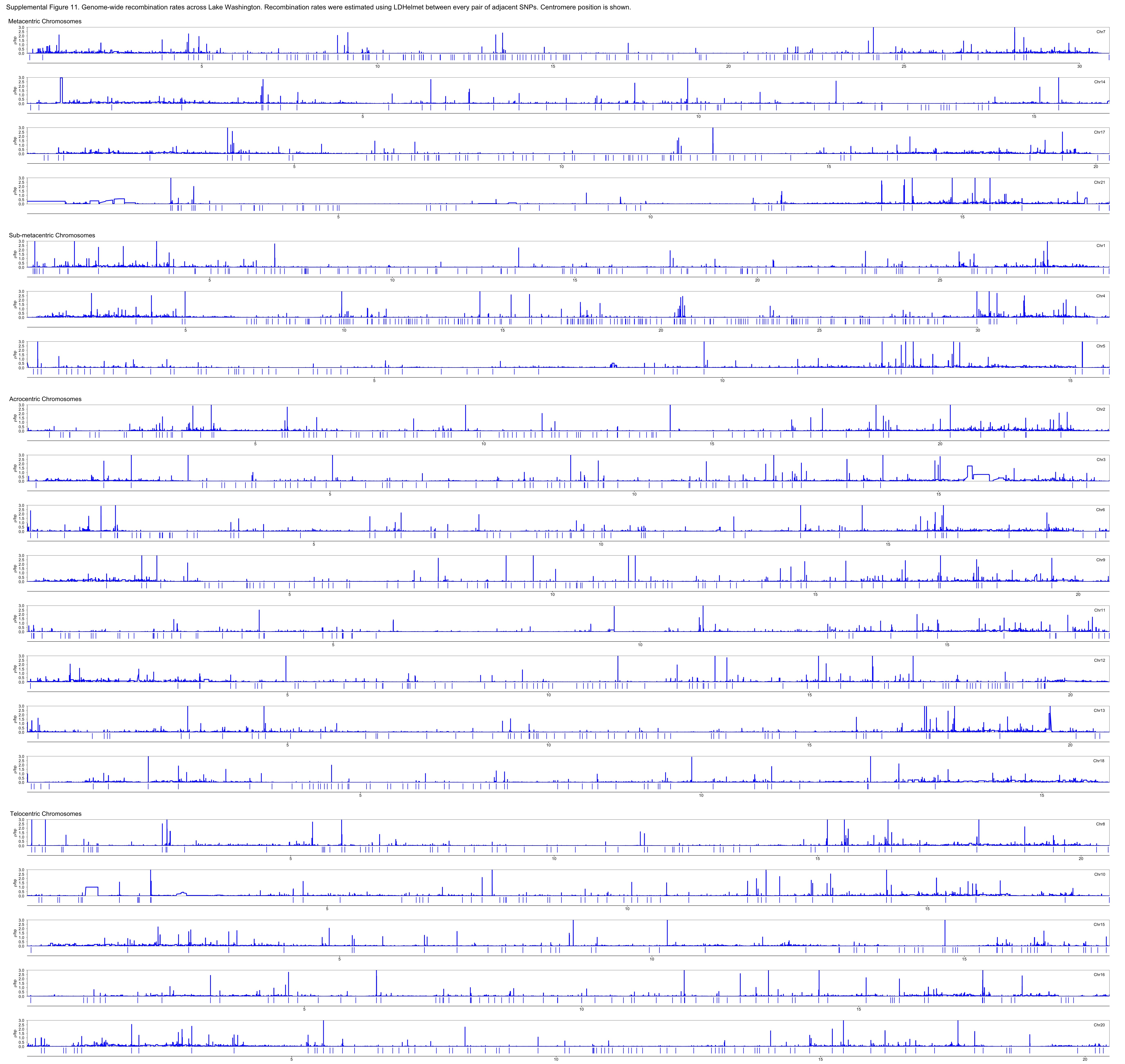

Supplement: Supplementary_Material_evz090 [file supplementary_material_evz090.zip › SuppFig6_GBE_revision_final_march.jpeg]

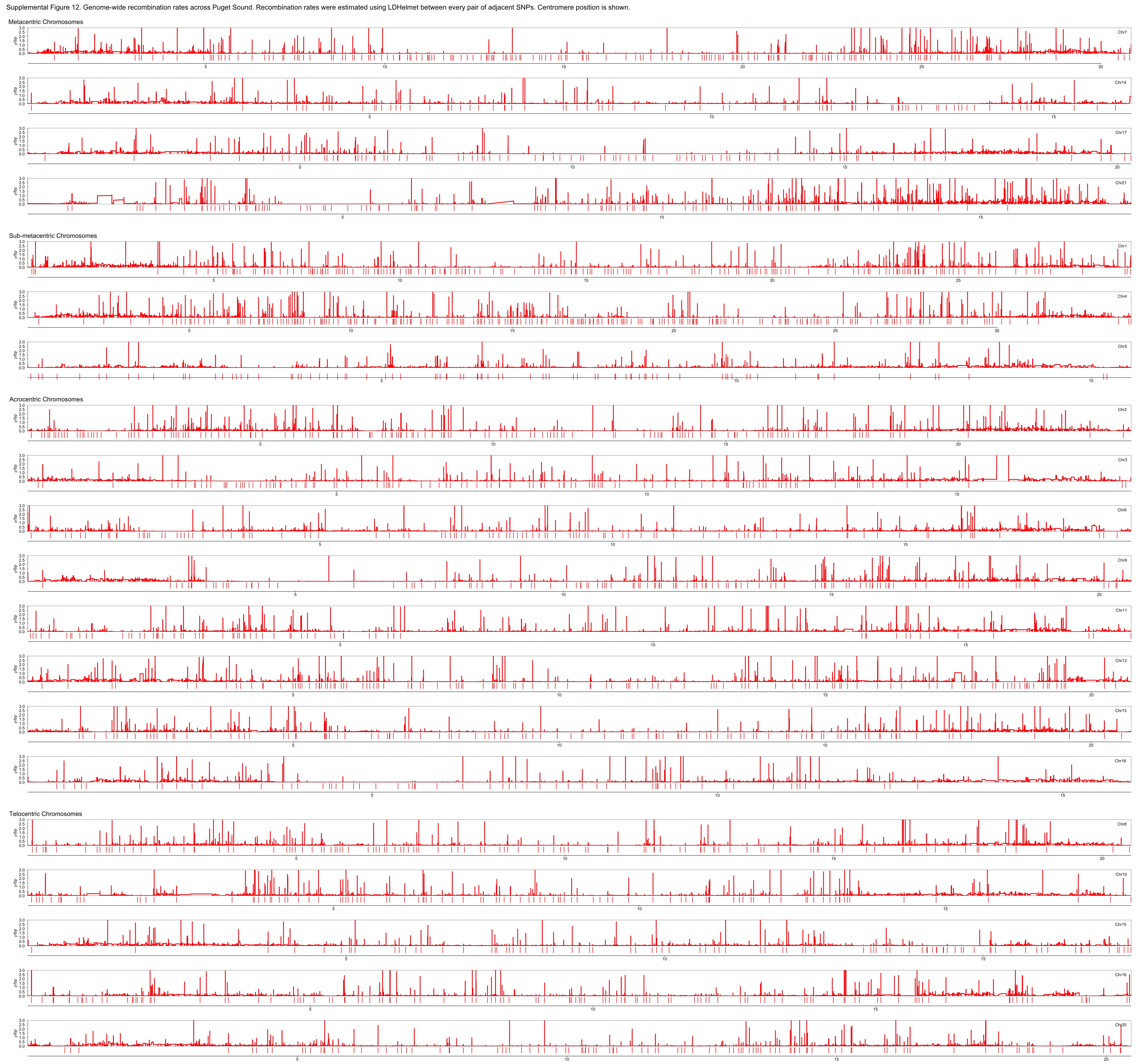

Supplement: Supplementary_Material_evz090 [file supplementary_material_evz090.zip › SuppFig7_GBE_revision_final_march.jpeg]
